# Super-enhancer-driven MLX mediates redox balance maintenance via SLC7A11 in osteosarcoma

**DOI:** 10.1038/s41419-023-05966-y

**Published:** 2023-07-17

**Authors:** Weitang Guo, Xin Wang, Bing Lu, Jiaming Yu, Mingxian Xu, Renxuan Huang, Mingzhe Cheng, Meiling Yang, Wei Zhao, Changye Zou

**Affiliations:** 1 Department of Musculoskeletal Oncology, The First Affiliated Hospital of Sun Yat-sen University, Guangzhou, 510080 China; 2 CAS Key Laboratory of Quantitative Engineering Biology, Shenzhen Institute of Synthetic Biology, Shenzhen Institute of Advanced Technology, Chinese Academy of Sciences, Shenzhen, 518055 China; 3 Guangdong Provincial People’s Hospital, Guangdong Academy of Medical Sciences, Guangzhou, 510080 China; 4 Key Laboratory of Stem Cells and Tissue Engineering (Sun Yat-Sen University), Ministry of Education, Guangzhou, 510080 China

**Keywords:** Sarcoma, Paediatric cancer, Cancer metabolism, Cancer epigenetics

## Abstract

Osteosarcoma (OS) is a common type of bone tumor for which there has been limited therapeutic progress over the past three decades. The prevalence of transcriptional addiction in cancer cells emphasizes the biological significance and clinical relevance of super-enhancers. In this study, we found that Max-like protein X (MLX), a member of the Myc-MLX network, is driven by super-enhancers. Upregulation of MLX predicts a poor prognosis in osteosarcoma. Knockdown of *MLX* impairs growth and metastasis of osteosarcoma in vivo and in vitro. Transcriptomic sequencing has revealed that MLX is involved in various metabolic pathways (e.g., lipid metabolism) and can induce metabolic reprogramming. Furthermore, knockdown of *MLX* results in disturbed transport and storage of ferrous iron, leading to an increase in the level of cellular ferrous iron and subsequent induction of ferroptosis. Mechanistically, *MLX* regulates the glutamate/cystine antiporter SLC7A11 to promote extracellular cysteine uptake required for the biosynthesis of the essential antioxidant GSH, thereby detoxifying reactive oxygen species (ROS) and maintaining the redox balance of osteosarcoma cells. Importantly, sulfasalazine, an FDA-approved anti-inflammatory drug, can inhibit SLC7A11, disrupt redox balance, and induce massive ferroptosis, leading to impaired tumor growth in vivo. Taken together, this study reveals a novel mechanism in which super-enhancer-driven *MLX* positively regulates *SLC7A11* to meet the alleviated demand for cystine and maintain the redox balance, highlighting the feasibility and clinical promise of targeting SLC7A11 in osteosarcoma.

## Introduction

Osteosarcoma (OS) is the most common type of malignant bone tumor, often occurring in adolescents. In the 1970s, the clinical application of chemotherapy drugs combined with surgical resection significantly improved the five-year survival rate of patients with OS [1]. However, few therapeutic advances have been made since then to further improve the clinical outcomes of OS patients [2]. Whole-genome and exome sequencing of OS have revealed the genomic landscape of OS and highlighted the prevalence of chromothripsis [3], a phenomenon whereby simultaneous chromosomal rearrangements in certain genomic regions generate a large number of genomic mutations in OS patients. In addition to these large-scale genomic rearrangements, some recurrent genetic alterations involving *TP53*, *RB1*, *ATRX*, and *DLG2* have been identified [4]. However, the identification of these genomic alterations has failed to lead to successful clinical translation, thus necessitating the discovery of more druggable targets.

Enhancers, which are cis-regulatory elements localized distal to promoters and transcription start sites, play a critical role in determining cell identity by regulating the transcription of downstream genes [5]. Recently, super-enhancers, a long stretch of genomic region comprising one or more enhancers in close proximity, have been reported to have a significant impact on cell fate determination [6]. The capacity to control cell identity makes super-enhancers appealing targets for exploitation by cancer cells to transform and maintain their phenotypes [7]. Super-enhancers can be hijacked by cancer cells to promote early progression [8, 9] and metastasis [10]. Recently, we found that the covalent CDK7 inhibitor THZ2 can disrupt the transcription of super-enhancer-associated oncogenes and exhibited promising anti-osteosarcoma effects both in vitro and in vivo [11]. Additionally, we discovered that inhibition of core regulatory circuitries reduces transcriptional activities at super-enhancer loci, thereby suppressing metastasis and chemoresistance in osteosarcoma [12], which makes super-enhancers a promising class of drug targets in osteosarcoma.

Metabolic reprogramming is a hallmark of cancer development. The atypically high demand for various metabolites (e.g., amino acids and nucleosides) required by the rapid proliferation of cancer cells is not just a side effect of abnormal mitochondrial function, but rather a consequence of metabolic reprogramming directed by oncogenes. For example, Myc is a key transcription factor that is frequently amplified in numerous malignant tumors including osteosarcoma [13]. The direct involvement of Myc in mitochondrial biogenesis, glutamine uptake, and amino acid synthesis further emphasizes its importance in shaping metabolism [14]. Mechanistically, Myc forms a dimer with MAX and binds to the E-box (CANNTG) motif to activate transcription of its target genes. In addition to acting in coordination with Max, Myc is also a part of the Max-like protein X (Max-MLX) network consisting of several highly conserved bHLH transcription factors. The Myc-MLX network has been implicated in a wide spectrum of biological processes, including cellular homeostasis and cancer progression [15–17]. Given the importance of *MYC* in cancer progression, it has long been recognized as a drug target despite the infeasibility of targeting Myc. Interestingly, super-enhancers have been identified as key regulators of *MYC* gene expression, providing a new route for targeting Myc [18, 19]. This targeting strategy has yielded clinical benefits, especially for hematological malignancies [20]. However, basic research and drug development have largely centered on Myc and overlooked other members of the Myc-MLX network, which may lead to novel drug targets being missed.

In this study, we conducted a genome-wide super-enhancer analysis and found that MLX, a central node of the Myc-MLX network, is also driven by super-enhancers. In addition, we found that the expression level of MLX is significantly higher in osteosarcoma and is associated with a poor prognosis. In vitro and in vivo studies have shown that MLX plays an important role in tumor growth and metastasis. Transcriptomic analysis revealed that SLC7A11, a gene encoding a highly specific amino acid transporter for cysteine and glutamate, was significantly downregulated upon *MLX* knockdown. Furthermore, MLX binds directly to E-box-containing enhancers upstream of SLC7A11. Subsequently, knockdown of *MLX* has been reported to decrease cellular cysteine levels, deplete glutathione (GSH), and elevate reactive oxygen species (ROS), thus leading to ferroptosis. Defects in cell growth, invasion, and disrupted cysteine metabolism caused by *MLX* knockdown can be restored by overexpressing SLC7A11. More importantly, sulfasalazine (SAS), an FDA-approved anti-inflammatory agent targeting SLC7A11, induces massive lipid peroxidation and results in a significant decrease in tumor volume in vivo. In conclusion, our results suggest a novel mechanism whereby super-enhancer-driven *MLX* rewires cysteine metabolism in osteosarcoma cells through *SLC7A11* to cope with oxidative stress, which holds promise for therapeutic development in osteosarcoma.

## Materials and methods

### Osteosarcoma specimens and bone tissue collection

Surgical specimens of osteosarcoma and normal bone tissue were collected at the Department of Osteo-Oncology, The First Affiliated Hospital of Sun Yat-sen University in accordance with an institutionally approved protocol. Written informed consent was obtained from each study participant after a thorough explanation of the procedures and their risks, in accordance with the Declaration of Helsinki. All specimens were examined by a pathologist to verify tumor type and grade.

### Cell culture

Bone marrow mesenchymal stem cells (BMSCs) and human osteosarcoma cell lines 143B, SJSA1, HOS, ZOS, ZOSM, MG63, U2OS, and SAOS-2 were cultured in DMEM medium supplemented with 10% fetal bovine serum (FBS; BI, USA; Corning, USA) and 1% penicillin/streptomycin (Sigma, Munich, Germany) at 37 °C in a humidified 5% CO_2_ atmosphere.

### Cell viability assay

The activity of cell proliferation was assessed using the CellTiter-Glo® Luminescent Cell Viability Assay (G7570, Promega). Briefly, 100 μl of cell suspension containing less than 30,000 cells was seeded into each well of a 96-well plate suitable for chemiluminescence detection. The culture medium without cells was used as a negative control. The cells were cultured according to standard cell culture protocols, and if required, drug-treated cells were included. The drugs used in this study included Sulfasalazine/SAS (HY-14655), Liproxstatin-1 (HY-12726), Ferrostatin-1 (HY-100579), Deferoxamine/DFO (HY-B0988), Deferasirox (HY-17359), and Dimethyl sulfoxide/DMSO (HY-Y0320), all purchased from MCE.

### Invasion assay

143B and SJSA1 cells were seeded at the density of 2 × 10^4^ cells/200 μl in each Transwell® cell culture insert (8 μm pore size, 6.5 mm diameter; Costar, Cambridge, MA, USA) and allowed to migrate overnight. The Transwell® inserts were filled with 800 μl of Dulbecco’s modified Eagle’s medium supplemented with 10% fetal bovine serum. After incubation at 37 °C overnight, the filters were rinsed with PBS, fixed with 4% PFA (10 min), and stained with crystal violet (0.1%) for 30 min. The cells on the upper surface of the filters were removed with a cotton swab, and the cells that migrated through the filters were counted under the microscope at a magnification of 100×. Each clone was tested in triplicate in at least two independent assays. The data are presented as mean ± SD.

### Orthotopic xenograft model

A single-cell suspension containing 0.5-1 × 10^6^ osteosarcoma cells in 20 μl was injected into the right tibial medulla of 5-week-old nude mice that were anesthetized with chloral hydrate. After 10 days, osteosarcoma growth in vivo was analyzed using an in vivo imaging system (IVIS; Xenogen) while the mice were under isoflurane anesthesia.

### Patient-derived xenograft model

Tumor specimens were obtained from osteosarcoma patients who provided informed consent. During surgery, tumor fragments were collected and kept on ice for less than 50 minutes. Fresh tumor fragments were subcutaneously transplanted into the upper back subcutaneous tissue of 5-week-old NCG (NOD-Prkdcem26Il2rgem26/Nju) mice under 10% chloral hydrate anesthesia. Xenografts appeared at the graft site 1 to 3 months after transplantation, and were subsequently transplanted from mouse to mouse. Animals without tumor growth were excluded, and the remaining animals were randomly divided into control and treatment groups, ensuring equal initial tumor size distribution. Tumor volumes were calculated using the formula: volume (mm^3^) = (length × width × width) / 2. Once the tumors reached 20–50 mm^3^ in volume, the mice were randomly assigned to different treatment groups for either sulfasalazine or saline. Tumor size was measured every 2 days using calipers to record length and width. After the mice were sacrificed, tumor xenografts were removed, fixed in formalin, and stored at 4 °C. Animal experiments were approved by the Animal Care and Use Committee of Sun Yat-sen University. Sulfasalazine was purchased from Master of Bioactive Molecules.

### shRNA and overexpression assay

shRNA sequences specific for target genes were designed and cloned into pLKO puro lentiviral vectors. To generate lentiviral particles, the constructed shRNA expression plasmids were co-transfected into human embryonic kidney 293 T cells with the packaging plasmids pVSVg and psPAX2 using liposomal amine 2000 (Invitrogen, USA). Osteosarcoma cells 143B and SJSA1 were infected with the obtained lentivirus to knock down the target gene. 143B cells stably expressing *SLC7A11* and control cells were infected with pCDH-*SLC7A11* and empty pCDH, respectively. The sequences of shRNA are listed in Table S1, Supporting Information.

### RNA-seq and data analysis

Cells were lysed in TRIzol reagent and total RNA was extracted using the RNeasy Mini Kit (QIAGEN, 74106) according to the user’s manual and quantified using Nanodrop 2000. RNA integrity was analyzed using an Agilent Bioanalyzer 2100. RNA-seq libraries were constructed using Illumina TruSeq RNA library prep kit V2 according to the manufacturer’s manual. Paired-end sequencing was carried out using Illumina HiSeq 2500 platform. The resulting reads were first proceeded for quality control using FastQC v0.11.9. RNA-seq reads were then mapped to the human genome hg38 using STAR 2.7.9a [21]. Reads counting was performed using HTSeq [22] and the R package DESeq2 [23] was used for transcripts quantification and differentially expressed gene analysis (padj < 0.05 as cutoff). Gene Ontology (GO) analysis and Gene Set Enrichment Analysis (GSEA) were performed using ClusterProfiler [24] and GSEA [25], respectively.

### Chromatin immunoprecipitation (ChIP) and ChIP-qPCR

Cells were fixed with 1% formaldehyde and subsequently quenched with glycine. After washing with PBS, the fixed samples were suspended in cell lysis buffer (5 mM PIPES-KCl pH 8.0, 85 mM KCl, 0.5% NP40), and collected nuclei were stored at −80 °C as nuclear pellets or nuclear lysates dissolved in nuclei lysis buffer (50 mM Tris-HCl pH8.0, 10 mM EDTA, 1% SDS). For MLX ChIP, lysates were thawed and sonicated with Sonifier (BRANSON) to obtain chromatin fragments of 300–1,000 bp, and 10-fold-diluted with ChIP dilution buffer (16.7 mM Tris-HCl pH8.0, 1.2 mM EDTA, 0.01% SDS, 1.1% Triron100×, 167 mM NaCl). After double-dilution with a sonication buffer (90 mM Hepes pH 7.9, 220 mM NaCl, 10 mM EDTA, 1% NP 40, 0.2% sodium deoxycholate, 0.2% SDS), nuclei were homogenized with a Bioruptor (Tosho denki). After washing, a complex of nuclear proteins/DNAs and antibodies against MLX (Cell Signaling technology; D8G6W; 1:100 dilution) was retrieved with Protein A- and Protein G-Dynabeads (Life Technologies). After the cross-linking was reversed, chromatin fragments were treated with RNase A and proteinase K. DNA was purified with phenol-chloroform extraction or Ampure XP (Beckman Coulter). In the ChIP-qPCR analyses, the values from the immunoprecipitated samples were normalized to input DNA. Primer sequences are listed in Table S2, Supporting Information.

### ChIP-seq and super-enhancer analysis

ChIP-seq was done as previously described [12]. Antibodies used for MLX and H3K27ac were anti-MLX (85570 S, Cell Signaling Technology) and anti-H3K27ac (07-360, Millipore), respectively. As for data analysis, FastQC was used for examining the quality of sequenced reads. Reads were then aligned to the human genome hg38 using bowtie2 and duplicated reads were then removed using samtools [26]. Identification of peaks is carried out by utilizing the “broad peak” calling mode of MACS3 [27]. DeepTools [28] was used to create bigwig files and Integrative Genomics Viewer (IGV) [29] was used for ChIP-seq visualization. Super-enhancer calling was carried out using Ranking of Super Enhancers [6] and the super-enhancer-associated genes were identified using GREAT [30].

### Western blot (WB)

Cell lines or tumor tissues were harvested and lysed in RIPA buffer (1% NP 40, 0.5% sodium deoxycholate, 0.1% SDS, 10 ng/ml PMSF, 0.03% aprotinin, and 1 μM sodium orthovanadate) on ice for 30 min. After centrifugation for 15 min at 14,000 rpm, the supernatants were collected, and the protein concentration was quantified by using Bradford assay. The proteins were separated on 12% SDS-PAGE gels and transferred to polyvinylidene difluoride membranes. The membranes were blocked with 5% skim milk and incubated with primary antibodies, such as MLX (12042-1-AP, Proteintech), β-actin (81115-1-RR, Proteintech), TRC/CD71 (66180-1-Ig, Proteintech), SLC40A1/FPN1 (26601-1-AP, Proteintech), Transferrin (66171-1-Ig, Proteintech), FTH1 (3998S, Cell Signaling Technology), cleaved Capase-3 (9661S, Cell Signaling Technology), SLC7A11(12691S, Cell Signaling Technology), Tubulin (ab176560, Abcam) and 4-Hydroxynonenal (ab46545, Abcam), Caspase-3 (ab184787, Abcam), and corresponding horseradish peroxidase-conjugated secondary antibodies were used against each primary antibody. Proteins were detected and filmed by using chemiluminescent detection reagents.

### Immunohistochemistry (IHC) assay

The formalin-fixed and paraffin-embedded tumor tissue sections were dewaxed with xylene and graded ethanol, and then rinsed with water. Then, the paraffin sections were heated for 30 minutes with Tris/EDTA (pH = 9.0) buffer in a microwave oven and cooled at room temperature for antigen repair. The slides were then incubated in PBS with 0.05% TritonX100 for 5 minutes. After quenching the endogenous hydrogen peroxide with PBS containing 3% H_2_O_2_, the slides were treated continuously in a humidification chamber. The slides were sealed with goat serum at room temperature for 1 hour, then incubated with primary antibodies at 4 °C overnight. The slides were then incubated with secondary rabbit antibodies at room temperature for 1 hour. DAB staining was performed for 1–3 minutes at room temperature, followed by reverse staining with hematoxylin and cover slides. The expression of MLX was evaluated according to the staining intensity and the percentage of positive cells. Staining intensity was scored as follows: 0 for negative, 1 for weak positive, 2 for moderate positive, and 3 for strong positive. The percentage of positive cells was scored as follows: 0 for completely negative, 1 for 25% positive, 2 for 26–50% positive, and 3 for 51–75% positive, and 4 for 76–100% positive. The final score was calculated by adding the scores for staining intensity and the percentage of positive cells. Scores between 0–3 were considered as low expression and scores higher than 3 were considered as high expression. Other antibodies were used for IHC staining as shown: Ki-67 (27309-1-AP, Proteintech), MLX (12042-1-AP, Proteintech), SLC7A11 (12691S, Cell Signaling Technology), and 4-HNE (ab46545, Abcam). Cells with positive staining in the cell membrane were counted under a light microscope at ×400 magnification.

### Hematoxylin-eosin (HE) staining

After routine dewaxing, rinse the slices with double distilled water and stain with hematoxylin for 8 minutes. The slices should then be soaked in distilled water for 30 seconds, followed by 95% ethanol and staining with eosin for 1 minute. Alcohol, xylene, and neutral glue should be used for dehydration, transparency, and sealing, respectively. The slices should then be imaged using a microscope with ×400 magnification.

### Lipid peroxidation measurement

Inoculate the cells in a 6-well plate and culture them overnight. The next day, harvest the cells by trypsin digestion and suspend them in 200 μl of PBS containing 5 μM C11-BODIPY581/591 (Invitrogen). Incubate the cells at 37 °C in a water bath for 30 minutes. Assess lipid peroxidation using the flow cytometer BD Accuri C6 with a 488 nm laser on an FL1 detector. Analyze a minimum of 10,000 single cells per well.

### Glutathione (GSH) measurement

4 × 10^5^ cells expressing sh*MLX* per well were seeded into 6-well dishes and cultured for 24 hours. Then, cells were collected by trypsin digestion and prepared for measurement of glutathione using the Glutathione Assay Kit Instructions (A006-2-1, Nanjing Jiancheng) according to the user guide.

### Determination of ROS generation

Changes in intracellular ROS levels were determined by measuring the oxidative conversion of cell permeable 2′,7′-dichlorofluorescein diacetate (DCFH-DA) to fluorescent dichlorofluorescein (DCF) in fluorospectro-photometer (F4000, Japan). Cells were cultured in 6-well plates overnight and washed by PBS. Then, cells were collected and incubated with DMEM with 10 μM DCFH-DA at 37 °C for 20 min. Then DCF fluorescence distribution of 4 × 10^6^ cells was detected by fluorescence microplate reader at an excitation wavelength of 488 nm and at an emission wavelength of 535 nm.

### Cell ferrous iron (Fe^2+^) measurement

Intracellular ferrous iron levels were measured using the Cell Ferrous Iron Colorimetric Assay Kit (E-BC-K881-M, elabscience, China) and a fluor spectrophotometer (F4000, Japan). Cells were seeded overnight in 6-well plates and washed with PBS. The collected cells (1 × 10^6^) were processed according to the manufacturer’s instructions, and the supernatant was collected by centrifugation. Then, 80 μl of supernatant was added to a 96-well enzyme plate, and the OD value of each well was measured at 593 nm after adding the reaction reagent.

### Quantitative and statistical analyses

GraphPad Prism version 8.0 (GraphPad Software, USA) was used for all analyses. Student’s t-test was used to compare individual data points between two groups. Unpaired or paired two-tailed t-tests were used to compare the statistical significance of all tests. Comparisons among three or more groups were performed using one-way ANOVA test. Pearson correlation coefficient was calculated to show the relationship between the expression levels of MLX and SLC7A11 protein. The Kaplan–Meier method and the log-rank test were used to compare patient survival. Data are presented as mean ± SD. In all analyses, p < 0.05 was considered to indicate statistical significance. *p < 0.05; **p < 0.01; ***p < 0.001 is based on the Student’s t-test; n.s., non-significant.

## Results

### MLX is driven by super-enhancers in osteosarcoma and correlated with poor prognosis

**Fig. 1:**
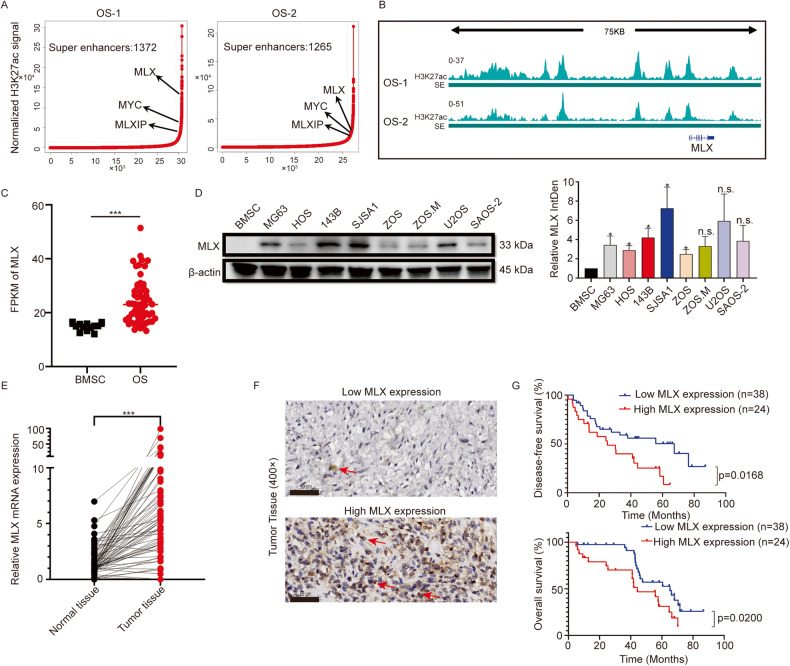
Upregulation of SE-driven *MLX* in osteosarcoma is correlated with poor prognosis. **A** Stitched enhancers ranked by H3K27ac ChIP-seq signal in two clinical samples. **B** The H3K27ac signal near the upstream super-enhancers of MLX locus. **C** Transcriptomic sequencing results showing the expression of MLX in OS and BMSC from publicly available datasets [33–35]. Data are represented as mean ± SD, BMSC, n = 12; OS, n = 60. Unpaired t-test was used. **D** Western blot analysis of MLX in various OS cell lines and BMSC. Data are represented as mean ± SD, n = 3. Unpaired t-test was used. **E** RT-qPCR analysis of relative *MLX* expression in osteosarcoma tissues and adjacent normal tissues. Data are represented as mean ± SD, n = 72. Paired t-test was used. ***: *P* ≤ 0.001, **: *P* ≤ 0.01, *: *P* ≤ 0.05, n.s.: not significant. **F** Representative IHC staining of OS tissues with low- or high-MLX (scale bar = 50 μm). **G** Kaplan–Meier analysis of overall and disease-free survival for osteosarcoma patients with high- or low-MLX expression.

To identify the key oncogenes driven by super-enhancers in osteosarcoma, we performed chromatin immunoprecipitation-coupled high-throughput sequencing (ChIP-seq) for H3K27ac using clinical samples of osteosarcoma, and the resulting data were used for super-enhancer analysis [6]. We identified the Myc-MLX network members *MLX*, *MLXIP*, and *MYC* as super-enhancer-associated genes in both clinical samples (Fig. 1A). Furthermore, ChIP-seq binding profiles of these two patients and three publicly available osteosarcoma patients [31] showed that *MLX*-associated super-enhancer sites were enriched in active histone modification marks such as H3K27ac (Fig. 1B and Supplementary Fig. S1a). MLXIP/MondoA, the binding partner of MLX, was reported to be indispensable for the tumorigenesis of MYC-amplified tumors, demonstrating the oncogenic role of *MLXIP* [32]. Here, we sought to uncover the role of *MLX*, another member of the Myc-MLX network, in osteosarcoma. To investigate the expression of *MLX* in osteosarcoma, we first compared transcriptome sequencing data from the public osteosarcoma database [33–35] and we found that MLX expression was markedly higher in osteosarcoma tissues than in bone marrow mesenchymal stem cells (BMSCs) (Fig. 1C). To confirm the upregulation of *MLX*, we examined the expression of *MLX* in osteosarcoma cell lines, and we found that MLX was highly expressed in osteosarcoma cell lines compared to BMSCs. Moreover, highly invasive cell lines with more aggressive tumor phenotypes, such as 143B and SJSA1, expressed higher levels of *MLX* (Fig. 1D). To verify the results in clinical samples, we conducted RT-qPCR of 72 pairs of osteosarcoma samples, and the results showed significantly higher expression of *MLX* in osteosarcoma compared with paired normal tissues (Fig. 1E). Additionally, we performed immunohistochemical staining for MLX on 62 paraffin sections of osteosarcoma tissues and evaluated their pathological scores. Kaplan–Meier analysis of the aforementioned clinical samples revealed that high MLX expression was correlated with poor overall survival and disease-free survival (Fig. 1F, G). Collectively, these findings indicate that upregulation of SE-driven *MLX* in osteosarcoma is correlated with poor prognosis.

### MLX knockdown significantly suppresses the growth and metastasis of osteosarcoma cells in vitro and in vivo

**Fig. 2:**
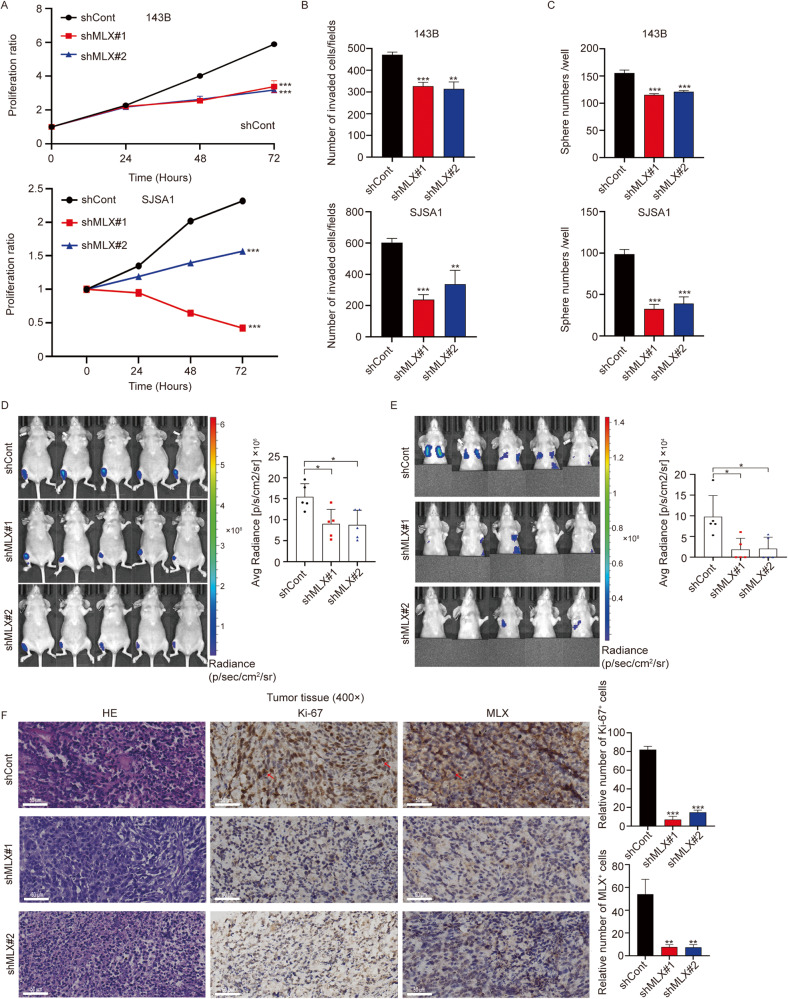
*MLX* is essential for the growth and metastasis of osteosarcoma cells in vitro and in vivo. **A** Relative growth curves of 143B and SJSA1 cells stably transduced with nontargeting scrambled control shRNA (shCont) or two *MLX* shRNAs (sh*MLX*#1 and sh*MLX*#2). Data are represented as mean ± SD, n = 3. Unpaired one-way ANOVA test followed by Dunnett’s test was used. **B** Invasion assay conducted in sh*MLX* versus shCont in 143B (upper panel) and SJSA1 (lower panel). Data are represented as mean ± SD, n = 3. Unpaired t-test was used. **C** Sphere formation assay in 143B (upper panel) and SJSA1 cells (lower panel). Data are represented as mean ± SD, n = 3. Unpaired t-test was used. **D** Representative images of tumor growth in 143B-derived orthotopic model (n = 5 per group). Unpaired t-test was used. **E** The representative images of metastasis in 143B-derived orthotopic model (n = 5 per group). Unpaired t-test was used. **F** Representative images of H&E, Ki-67 and MLX staining of 143B-shCont and 143B-sh*MLX* tumors and the respective analyses (scale bar = 50 μm). Data are represented as mean ± SD, n = 3. Unpaired t-test was used. ***: *P* ≤ 0.001, **: *P* ≤ 0.01, *: *P* ≤ 0.05, n.s.: not significant.

To further document the consequences of the disruption of *MLX* expression in osteosarcoma, the aggressive human osteosarcoma cell lines 143B and SJSA1 with high *MLX* expression were chosen for functional analysis of *MLX* knockdown. We stably suppressed *MLX* expression using two distinct shRNAs (sh*MLX*#1 and sh*MLX*#2, the sequences are shown in the Supplementary Materials) in these two cell lines (Supplementary Fig. S2a). First, we found that *MLX* silencing impaired cell proliferation, invasion, and sphere formation in 143B and SJSA1 cells (Fig. 2A–C and Supplementary Fig. S2b, c). To further investigate the functional role of *MLX* in vivo, luciferase-transduced 143B cells stably expressing shControl or sh*MLX* were orthotopically injected into the central cavity of the bones of immunocompromised mice. We then monitored the mice using a live imaging system and found that cells expressing sh*MLX* exhibited a significant reduction in tumor growth compared to the controls (Fig. 2D). Furthermore, *MLX* silencing significantly reduced the probability of lung metastasis in orthotopic tumors (Fig. 2E). Additionally, IHC staining revealed a decrease in Ki-67^+^ and MLX^+^ cell populations in 143B-sh*MLX* tumors compared to 143B-shControl tumors, thus confirming the role of MLX in promoting cell proliferation (Fig. 2F). In addition to cell proliferation, H&E and Ki67 staining revealed fewer metastases upon knockdown of *MLX* (Supplementary Fig. S2d). Collectively, these data suggest that *MLX* promotes osteosarcoma cell growth, invasion, and sphere formation in vitro, and *MLX* knockdown significantly suppresses the growth and metastasis of osteosarcoma in vivo.

### The MLX-SLC7A11 axis is involved in metabolic pathways

**Fig. 3:**
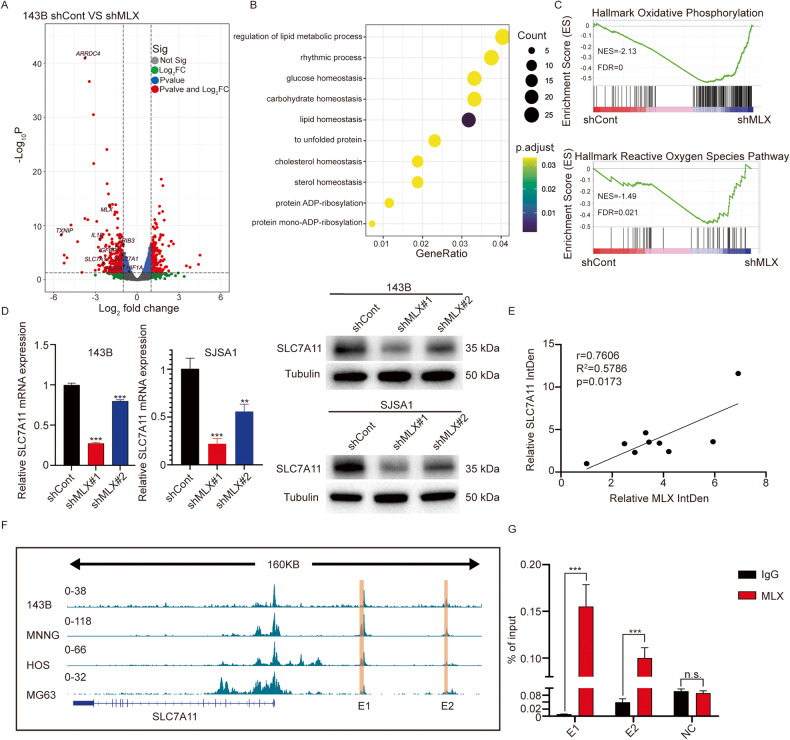
MLX is critical for metabolic activities and promotes *the expression of SLC7A11* through binding to its enhancers. **A** Analysis of differentially expressed genes in transcriptome sequencing (mRNA-seq) of 143B-sh*MLX*. **B** GO analysis of the significantly down-regulated genes upon *MLX* knockdown. **C** GSEA analysis of oxidative phosphorylation pathway and reactive oxygen species pathway. **D** The mRNA and protein level of SLC7A11 in 143B and SJSA1 upon *MLX* knockdown by RT-qPCR and WB. Data are represented as mean ± SD, n = 3. Unpaired t-test was used. **E** Pearson’s correlation scatter plot of the expression levels of MLX and SLC7A11 in human osteosarcoma cell lines. **F** The two enhancer sites containing E-box sequence upstream of SLC7A11 identified by ChIP-seq of H3K27ac [31] (GEO74230). **G** ChIP-qPCR analysis showing the occupancy of MLX at these two *SLC7A11* enhancer sites. Data are represented as mean ± SD, n = 4. Unpaired t-test was used. ***: *P* ≤ 0.001, **: *P* ≤ 0.01, *: *P* ≤ 0.05, n.s.: not significant.

To understand the mechanism underlying the tumorigenic function of *MLX* in osteosarcoma, we performed transcriptome sequencing (mRNA-seq) of *MLX*-silenced 143B cells (Supplementary Fig. S3a). Analysis of differentially expressed genes revealed that silencing *MLX* caused significant changes in gene expression (Fig. 3A). To determine the effects of *MLX* knockdown on biological processes, we performed GO analysis. The result showed that the target genes of *MLX* were linked to metabolic pathways, including the regulation of lipid metabolic processes, carbohydrate homeostasis, and lipid homeostasis, which was in agreement with the function of the Myc-MLX network (Fig. 3B). Furthermore, using GSEA, we found that oxidative phosphorylation (OXPHOS) and ROS pathways were significantly enriched in the *MLX* knockdown group, suggesting that MLX can reprogram the metabolic pathways related to the redox balance in osteosarcoma cells (Fig. 3C). Interestingly, we found that *SLC7A11*, a key player in intracellular redox balance, was significantly downregulated upon silencing of *MLX*, which was further validated by western blotting and RT-qPCR in 143B and SJSA1 cells (Fig. 3D). Osteosarcoma cell lines also exhibited elevated expression of *SLC7A11* (Supplementary Fig. S3b). Consistent with this observation, we also found a strong correlation between MLX and SLC7A11 in several cell lines (Fig. 3E). Given that MLX regulates the expression of *SLC7A11*, we next sought to investigate how MLX regulates *SLC7A11*. We examined the epigenetic landscape around the *SLC7A11* locus by analyzing H3K27ac (a histone modification signature of active TSS and enhancers) ChIP-seq data [31], and we identified two enhancer sites upstream of *SLC7A11*. Interestingly, these two enhancers contain E-box motifs, which can be bound by MLX, potentially allowing direct binding of MLX (Fig. 3F). To validate this, ChIP-qPCR analysis of *MLX* in 143B cells was carried out, and the results showed that MLX binds directly to these two enhancer sites (Fig. 3G), suggesting that MLX can directly regulate *SLC7A11* by binding to its enhancers. In conclusion, these results indicate that MLX may affect metabolic pathways (e.g., redox balance) in osteosarcoma cells by regulating *SLC7A11*.

### MLX knockdown induces cell death via ferroptosis in osteosarcoma cells

**Fig. 4:**
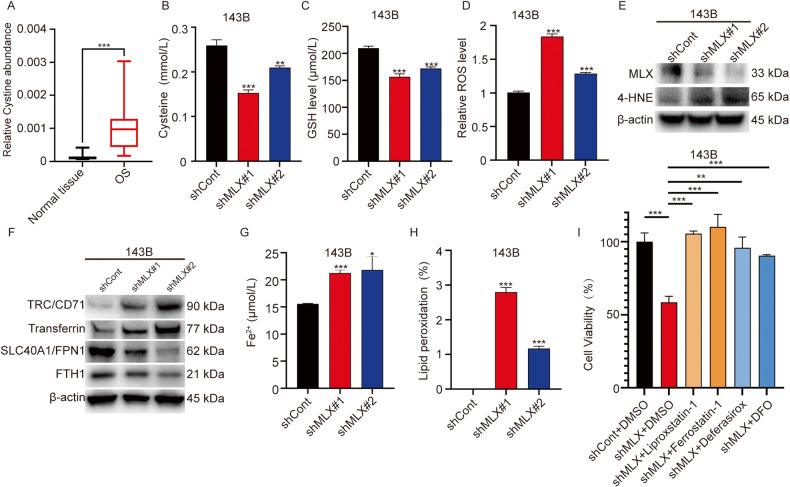
*MLX* knockdown disrupts redox balance and promotes ferroptosis in osteosarcoma cells. **A** Determination of cystine in 30 pairs of osteosarcoma and normal control tissues. Data are represented as mean ± SD. Paired t-test was used. **B**, **C** The measurement of Cysteine and GSH in 143B upon *MLX* knockdown. Data are represented as mean ± SD, n = 3. Unpaired t-test was used. **D** The measurement of ROS level in shCont- and sh*MLX*-143B. Data are represented as mean ± SD, n = 3. Unpaired t-test was used. **E** Detection of MLX and 4-HNE protein levels in 143B cells upon MLX knockdown by western blotting (WB). **F** WB analysis of transferrin, transferrin receptors, ferritin and ferroportin protein levels in 143B cells with sh*MLX* knockdown. **G** Measurement of intracellular ferrous iron levels in 143B cells upon *MLX* knockdown. Data are presented as mean ± SD, n = 3. Unpaired t-test was used. **H** Percentage of cells with increased levels of lipid peroxidation in shCont- and sh*MLX*-143B cells. Data are presented as mean ± SD, n = 3. Unpaired t-test was used. **I** 24-hour cell viability of osteosarcoma cells treated with *MLX* knockdown and ferroptosis inhibitors (10 μM Liproxstatin-1, 10 μM Ferrosatin-1, 50 μM Deferasirox, and 100 μM DFO). Data are presented as mean ± SD, n = 3. Unpaired t-test was used. ***: *P* ≤ 0.001, **: *P* ≤ 0.01, *: *P* ≤ 0.05, n.s.: not significant.

Given that *SLC7A11* is a key target gene of MLX and that *SLC7A11* encodes the cystine/glutamate transporter responsible for the export of glutamate and the uptake of cystine, we hypothesized that *MLX* may have an impact on cystine transport. To test this hypothesis, we performed metabolism studies of 30 paired tumor-normal samples, and we found a higher abundance of cystine in osteosarcoma compared to in normal tissue (Fig. 4A, Supplementary Table S3). Since intracellular cystine can be processed to cysteine, which is an important metabolic intermediate of glutathione (GSH) biosynthesis, we measured the levels of cysteine and GSH upon knockdown of *MLX*. As expected, we found lower levels of cysteine (Fig. 4B) and GSH (Fig. 4C) upon knockdown of *MLX*. As GSH is an important class of antioxidants that scavenges ROS in cells, we speculated that knockdown of *MLX* would lead to an increase in ROS levels. To test this, we measured ROS levels upon knockdown of *MLX* and found increased ROS levels in both *MLX*-silenced groups (Fig. 4D). It is worth noting that the increase of ROS in sh*MLX*#2 was not as prominent as that in sh*MLX*#1, and the less effective knockdown was concordant with the milder decrease in cysteine and GSH levels in sh*MLX*#2, confirming again that the generation of ROS is a consequence of decreased cysteine and GSH levels. It is widely accepted that ROS can cause programmed cell death, including apoptosis and ferroptosis. However, the mechanism by which *MLX* knockdown affects cell proliferation remains unclear. To address this, we first analyzed the cell cycle profile of *MLX*-silenced cells and ruled out the possibility of cell cycle arrest (Supplementary Fig. S4a). The activity of cleaved Caspase-3 and 4-HNE, which are markers of apoptosis and ferroptosis, respectively, were evaluated by western blotting. The results indicated that knockdown of *MLX* primarily induced ferroptosis rather than apoptosis, as there was no significant change in cleaved Caspase-3 (Supplementary Fig. S4b), but a substantial increase in 4-HNE (Fig. 4E). To further confirm that *MLX* knockdown does not affect apoptosis, apoptosis was measured using flow cytometry, but no significant apoptosis was observed upon *MLX* knockdown (Supplementary Fig. S4c). Moreover, nuclear translocation of AIF was not induced upon *MLX* knockdown (Supplementary Fig. S4d), indicating the absence of caspase-independent apoptosis. These findings suggest that cell growth defects upon *MLX* knockdown were not caused by apoptosis.

To examine whether ferroptosis was the main cause of cell growth defects in *MLX*-silenced cells, the levels of proteins related to intracellular iron transport were measured upon *MLX* knockdown (Fig. 4F). The results showed a significant increase in transferrin and transferrin receptors (TRC/CD71), indicating an accelerated influx of Fe^3+^. Additionally, a significant decrease in ferritin (FTH1) and ferroportin (SLC40A1/FPN1) was observed, which interferes with the storage and efflux of cellular ferrous iron. Therefore, the increase in iron inflow and the decrease in ferrous ion storage and outflow may lead to an accumulation of free ferrous iron in *MLX*-silenced cells. The increase in intracellular ferrous iron was confirmed by the quantification of lipid peroxidation, a direct cause of ferroptosis, upon silencing of *MLX* (Fig. 4G, H).

The application of lipid peroxidation inhibitors (liproxstatin-1 and ferrostatin-1) and iron chelators (deferasirox and deferoxamine/DFO) successfully rescued *MLX*-silenced osteosarcoma cells (Fig. 4I), verifying that knockdown of *MLX* could result in ferroptosis. Taken together, these results indicate that knockdown of *MLX* disrupts the GSH-ROS balance, causes accumulation of cellular ferrous iron, and induces lipid peroxidation, ultimately leading to ferroptosis.

### MLX shapes the redox balance through SLC7A11

**Fig. 5:**
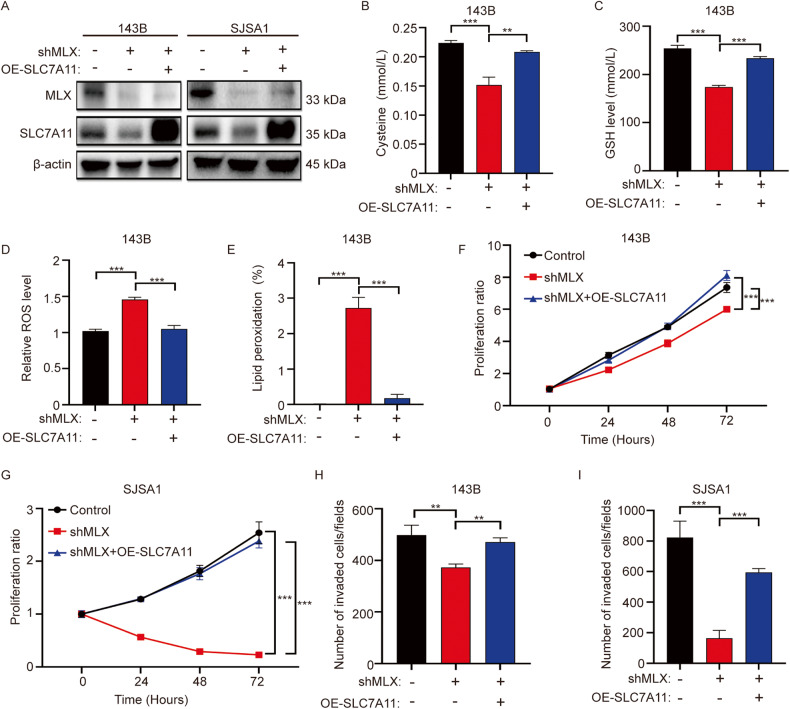
*SLC7A11* plays an important role in the growth and invasion of osteosarcoma cells and is the key downstream gene of MLX. **A** WB analysis of MLX and SLC7A11 in 143B (left) and SJSA1 (right) upon *MLX* knockdown. **B**–**E** The measurement of Cysteine, GSH, ROS and lipid peroxidation level upon overexpressing *SLC7A11* in *MLX*-silenced 143B cells. Data are represented as mean ± SD, n = 3. Unpaired t-test was used. **F**, **G** Relative growth curves of 143B (**F**) and SJSA1 (**G**) cells stably transduced with nontargeting scrambled control shRNA (shCont) or two *SLC7A11* shRNAs (sh*SLC7A11*#1 and sh*SLC7A11*#2). Data are represented as mean ± SD, n = 3. Unpaired one-way ANOVA test followed by Dunnett’s test was used. Cell invasion upon knockdown of sh*MLX* and/or overexpression *SLC7A11* in 143B (**H**) and SJSA1 (**I**) cells. Data are represented as mean ± SD, n = 3. Unpaired t-test was used. ***: *P* ≤ 0.001, **: *P* ≤ 0.01, *: *P* ≤ 0.05, n.s.: not significant.

To further verify the role of *SLC7A11* in mediating the function of MLX in shaping the redox balance, we knocked down *SLC7A11* in 143B and SJSA1 cells, and we discovered that knockdown of *SLC7A11* impaired both proliferative and invasive properties, phenocopying the knockdown of *MLX* (Supplementary Fig. S5a–d). More importantly, we overexpressed *SLC7A11* in *MLX*-silenced cells (Fig. 5A) and measured the levels of cysteine, GSH, ROS, and lipid peroxidation. The levels of cysteine and GSH were restored upon overexpression of SLC7A11 in *MLX*-silenced cells (Fig. 5B, C). Likewise, the levels of ROS and lipid peroxidation also decreased significantly upon knockdown of *MLX* but returned to the level in the control group following overexpression of *SLC7A11* (Fig. 5D, E), thus indicating that the observed ferroptosis in *MLX*-silenced cells was due to the decrease in GSH caused by downregulation of *SLC7A11*. In accordance with the changes in metabolites involved in redox balance, overexpression of *SLC7A11* restored the impaired cell proliferation and invasion resulting from *MLX* knockdown only, suggesting that normalization of the redox balance underlies the restored tumor aggressiveness (Fig. 5F–I and Supplementary Fig. S5e). Collectively, these results indicate that *SLC7A11* is indispensable for the redox-balancing function of MLX.

### The SLC7A11 inhibitor sulfasalazine exerts a powerful antitumor effect in vitro and in vivo

**Fig. 6:**
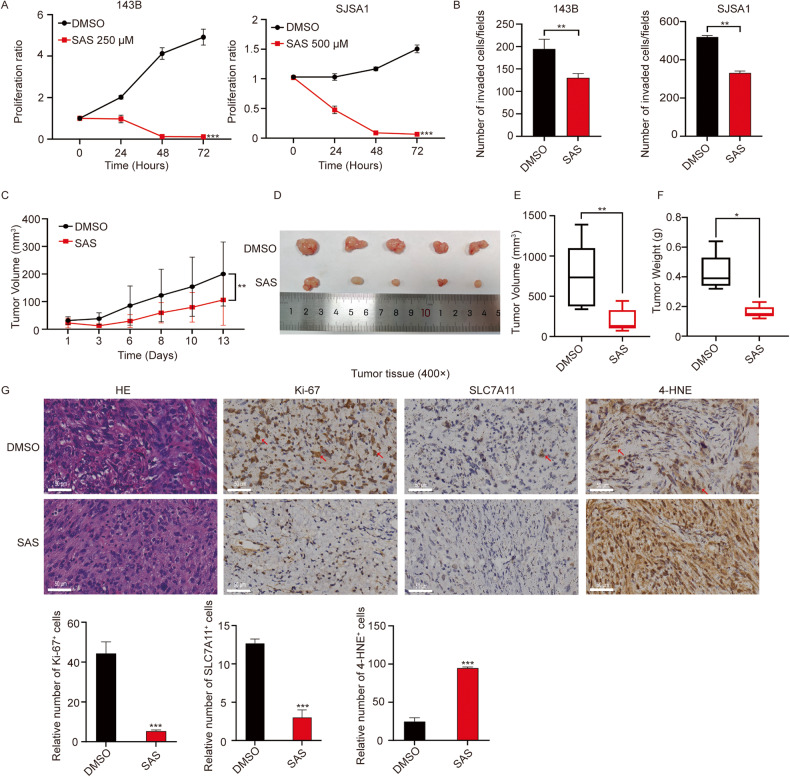
Sulfasalazine effectively suppresses the growth and metastasis of osteosarcoma in vitro and in vivo. **A** Relative growth curves of 143B (left) and SJSA1(right) cells treated with sulfasalazine (SAS) or DMSO. Data are represented as mean ± SD, n = 3. Paired t-test was used. **B** Analysis of cell invasion in 143B (left) and SJSA1 (right) upon treatment of SAS or DMSO. Data are represented as mean ± SD, n = 3. Unpaired t-test was used. **C** The PDX tumor growth curve upon treatment of SAS. Data are represented as mean ± SD, n = 5. Paired t-test was used. **D**–**F** Representative images of DMSO- or SAS-treated (100 mg/kg) PDX tissues on day 13 and the statistical analysis of PDX volume and weight. Data are represented as mean ± SD, n = 5. Unpaired t-test was used. **G** Representative images of H&E, Ki-67, SLC7A11 and 4-HNE staining of the PDX tissues and the respective statistical analysis (scale bar = 50 μm). Data are represented as mean ± SD, n = 3. Unpaired t-test was used. ***: *P* ≤ 0.001, **: *P* ≤ 0.01, *: *P* ≤ 0.05, n.s.: not significant.

After establishing the critical role of the MLX-SLC7A11 axis in metabolic reprogramming of osteosarcoma cells, we hypothesized that inhibiting this axis could disrupt the redox balance and cause ferroptosis, resulting in defects in the aggressive phenotypes of osteosarcoma. As direct MLX inhibitors are not available, we explored the possibility of targeting SLC7A11. We evaluated the feasibility of SAS, an FDA-approved SLC7A11 inhibitor used for treating inflammatory diseases, including rheumatoid arthritis and ulcerative colitis. We demonstrated through WB that sulfasalazine directly inhibited SLC7A11 in osteosarcoma cells, and this effect was enhanced with increasing concentrations (Supplementary Fig. S6a). We measured the IC50 of sulfasalazine in two aggressive osteosarcoma cell lines expressing high levels of MLX (Supplementary Fig. S6b), and found that sulfasalazine significantly suppressed the proliferation of both cell lines (Fig. 6A).

We also conducted experiments to assess the effects of SAS treatment on the levels of ROS, lipid peroxidation, and GSH in osteosarcoma cells. Our results showed that SAS treatment significantly decreased the levels of GSH, while increasing the levels of ROS and lipid peroxidation (Supplementary Fig. S6c–e), indicating that SAS treatment affected the redox balance of osteosarcoma cells. We evaluated the effect of ferroptosis inhibitors (ferrostatin-1 and liproxstatin-1) on the cell viability of 143B cells treated with SAS. The results indicated that the inhibitors rescued SAS-induced cell death (Supplementary Fig. S6f), confirming that SAS inhibited the proliferation of osteosarcoma cells through ferroptosis.

Furthermore, the results of Transwell® assays indicated that sulfasalazine compromised the invasive potential of the cells (Fig. 6B and Supplementary Fig. S6g). To assess the potential clinical relevance, we used a patient-derived xenograft (PDXs) model, which has been applied in preclinical drug testing. Continuous monitoring of PDXs showed that treatment with sulfasalazine greatly suppressed tumor growth, without affecting body weight (Fig. 6C–F and Supplementary Fig. S6h). Immunohistochemical analysis revealed that sulfasalazine markedly reduced the expression of the proliferative markers Ki-67 and SLC7A11 in the tumor tissues. Furthermore, the sulfasalazine-treated group had higher 4-HNE staining compared to the DMSO-treated group, indicating that ferroptosis induced by sulfasalazine could be the underlying cause of the growth reduction in PDXs (Fig. 6G). In summary, these results suggest that the SLC7A11 inhibitor sulfasalazine effectively suppressed the growth of osteosarcoma in vitro and in vivo, indicating the potential for clinical translation of sulfasalazine into osteosarcoma treatment.

## Discussion

**Fig. 7:**
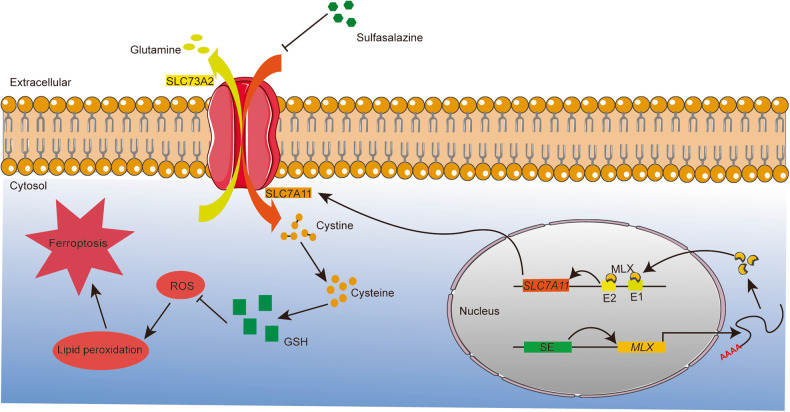
The scientific hypothesis of this study proposes that MLX expression is driven by SEs, and MLX in turn regulates the cystine/glutamate transporter SLC7A11, promoting cystine transport. Intracellular cystine is then converted to cysteine, which is involved in the synthesis of GSH. GSH plays a vital role in scavenging ROS and inhibiting lipid peroxidation, which is mediated by ROS and ultimately leads to ferroptosis. The pharmaceutical targeting of SLC7A11 with SAS induces ferroptosis in osteosarcoma cells. Further preclinical and clinical studies are needed to evaluate the potential of potent ferroptosis inducers, such as SAS, for osteosarcoma treatment.

In this study, we performed a genome-wide super-enhancer analysis and identified MLX, a member of the Myc-MLX network, as a super-enhancer-driven transcription factor. *MLX* is highly expressed in patients with osteosarcoma and plays an important role in promoting cell proliferation and invasion in vitro and in vivo. Mechanistically, MLX regulates the glutamate/cystine antiporter *SLC7A11* to promote the uptake of extracellular cystine for biosynthesis of the essential antioxidant GSH, thereby maintain the redox balance in osteosarcoma cells. Pharmacological inhibition of SLC7A11 with the FDA-approved anti-inflammatory drug sulfasalazine leads to ferroptosis and impairs the growth of xenografted osteosarcoma cells, highlighting the potential of targeting SLC7A11 in osteosarcoma treatment (Fig. 7).

It is widely accepted that gene dysregulation is a common feature of cancer. During development, gene expression is tightly regulated in order to establish different cell lineages. This lineage specification process is largely dependent on master transcription factors that bind to cell type-specific regulatory elements, known as enhancers. Super-enhancers, which are clusters of enhancers bound by master transcription factors, have recently been identified and reported to play a critical role in establishing cell identity [6, 7]. Because of their great potential to activate genes essential for establishing cell identity, super-enhancers are prone to being hijacked to transform normal cells into neoplastic cells [36]. The importance of super-enhancers in cancer initiation and progression has been established [37, 38], but the dependency on super-enhancers also offers a vulnerability for targeting. Targeting essential components of super-enhancers, such as *BRD4* and *CDK7*, has been shown to result in potent anti-tumor effects in preclinical and clinical studies [39–41]. In the present study, we identified *MLX* as a super-enhancer-driven gene and revealed its oncogenic role in osteosarcoma. Due to the absence of inhibitors targeting MLX, we were unable to test the effects of direct inhibition of MLX. However, several studies have shown that targeting components of super-enhancers attenuates tumor growth, confirming that osteosarcoma cells are addicted to super-enhancers and hence vulnerable to super-enhancer inhibitors [11, 42].

Metabolic reprogramming is another common feature of cancers. Altered metabolic pathways are not just a passive response to transformed cell identities but rather the result of oncogene-driven metabolic reprogramming, which also enhances the proliferative capacity of cancer cells [14]. As an example of oncogenes involved in metabolic reprogramming, *MYC* is well known for its varying amplification in numerous cancers, including osteosarcoma [13, 43]. *MYC* is involved in a wide range of biological processes by activating numerous genes [44]. Myc and MAX form a heterodimer and bind to the E-box sequence with high affinity to activate transcription [45]. Similar to MAX and Myc, MLX and MLXIP can also form a heterodimer to exert transcriptional regulation, forming an extended MYC regulatory network [15]. *MYC*-centered and *MLX*-centered networks crosstalk with each other and share a considerable degree of regulatory overlap [46]. Similar to *MYC*, *MLX* is involved in various metabolic pathways, including lipogenesis and glycolysis [47, 48]. In a recent study, MYCN was found to mediate cysteine addiction, and MYCN-amplified neuroblastoma cells were more prone to lipid peroxidation and ferroptosis when cysteine was depleted [49]. However, the involvement of MLX in cystine/cysteine-related metabolic pathways remains unclear, and in this study, we showed that MLX promotes the uptake of cystine via SLC7A11, and that knockdown of *MLX* induces lipid peroxidation and ferroptosis, which is consistent with the phenomenon observed in neuroblastoma. Considering that upregulation or amplification of MYC is a common event in osteosarcoma, the high demand for cystine might be at least partially met by MLX-mediated cystine uptake. However, exactly how MYC influences the function of MLX in osteosarcoma remains unclear and needs to be explored.

Ferroptosis, a form of programmed cell death, has provided new therapeutic opportunities for treating cancers refractory to conventional therapies. Studies have shown that the induction of ferroptosis can serve as an effective therapeutic method for triple-negative breast cancer [50] and renal cell carcinoma [51]. However, such studies have been very limited in osteosarcoma. In this study, we found that *SLC7A11* is highly expressed in osteosarcoma cells, suggesting a high demand for cystine. Targeting SLC7A11 has yielded promising results in hepatocellular carcinoma [52], breast cancer [53], and bladder cancer [54]. In our study, we used sulfasalazine, which is an FDA-approved SLC7A11 inhibitor that has been used to treat inflammatory diseases in the clinic, to trigger ferroptosis. The administration of sulfasalazine has been shown to enhance osteogenic differentiation of mesenchymal stem cells [55], and the progression of osteosarcoma is usually accompanied by defective osteogenic differentiation [56], suggesting that a greater osteogenic differentiation could result from the administration of sulfasalazine, which is beneficial for the treatment of osteosarcoma. Currently, only a few clinical trials have tested the effects of sulfasalazine in the treatment of brain (NCT04205357) and breast cancers (NCT03847311). Additional preclinical and clinical investigations are necessary to assess the efficacy of robust ferroptosis inducers such as sulfasalazine and sorafenib for the treatment of osteosarcoma.

In conclusion, we found that *MLX*, a member of the extended *MYC* family, is driven by super-enhancers. The high expression of *MLX* can promote uptake of cystine via *SLC7A11*, which fuels the biosynthesis of GSH to detoxify ROS and maintain the redox balance. Disruption of *MLX*-*SLC7A11* impairs cystine uptake, resulting in the accumulation of ROS, which in turn leads to lipid peroxidation, thereby triggering ferroptosis. Sulfasalazine, which is an FDA-approved drug, can be used to induce ferroptosis in PDXs, thereby highlighting the clinical potential of sulfasalazine in osteosarcoma treatment.

## Supplementary information

Supplemental material

TableS3_Cystine_OS

A reproducibility checklist

Full and uncropped western blots

## Data Availability

The transcriptome sequencing (mRNA-seq) of *MLX*-silenced 143B cells data generated during the current study are available in the Gene Expression Omnibus repository under accession number: GSE220173. The ChIP-seq and metabolomics data of human subjects that support the findings of this study are available from the corresponding author upon reasonable request.
