# Supplementary material for: Super-enhancer-driven MLX mediates redox balance maintenance via SLC7A11 in osteosarcoma
Source: Cell Death Dis. 2023 Jul 17;14(7):439. doi: 10.1038/s41419-023-05966-y (PMC10352384; doi:10.1038/s41419-023-05966-y)
Supplement: Supplementary file 1 — Supplemental material [file 41419_2023_5966_MOESM1_ESM.docx]

**Super-enhancer-driven MLX mediates redox balance maintenance via SLC7A11 in osteosarcoma**

Weitang Guo, Xin Wang, Bing Lu, Jiaming Yu, Mingxian Xu, Renxuan Huang, Mingzhe Cheng, Meiling Yang, Wei Zhao, Changye Zou

**Content**

1. **Supplemental Figures and legends**
2. **Supplemental Tables**

**Supplemental figures
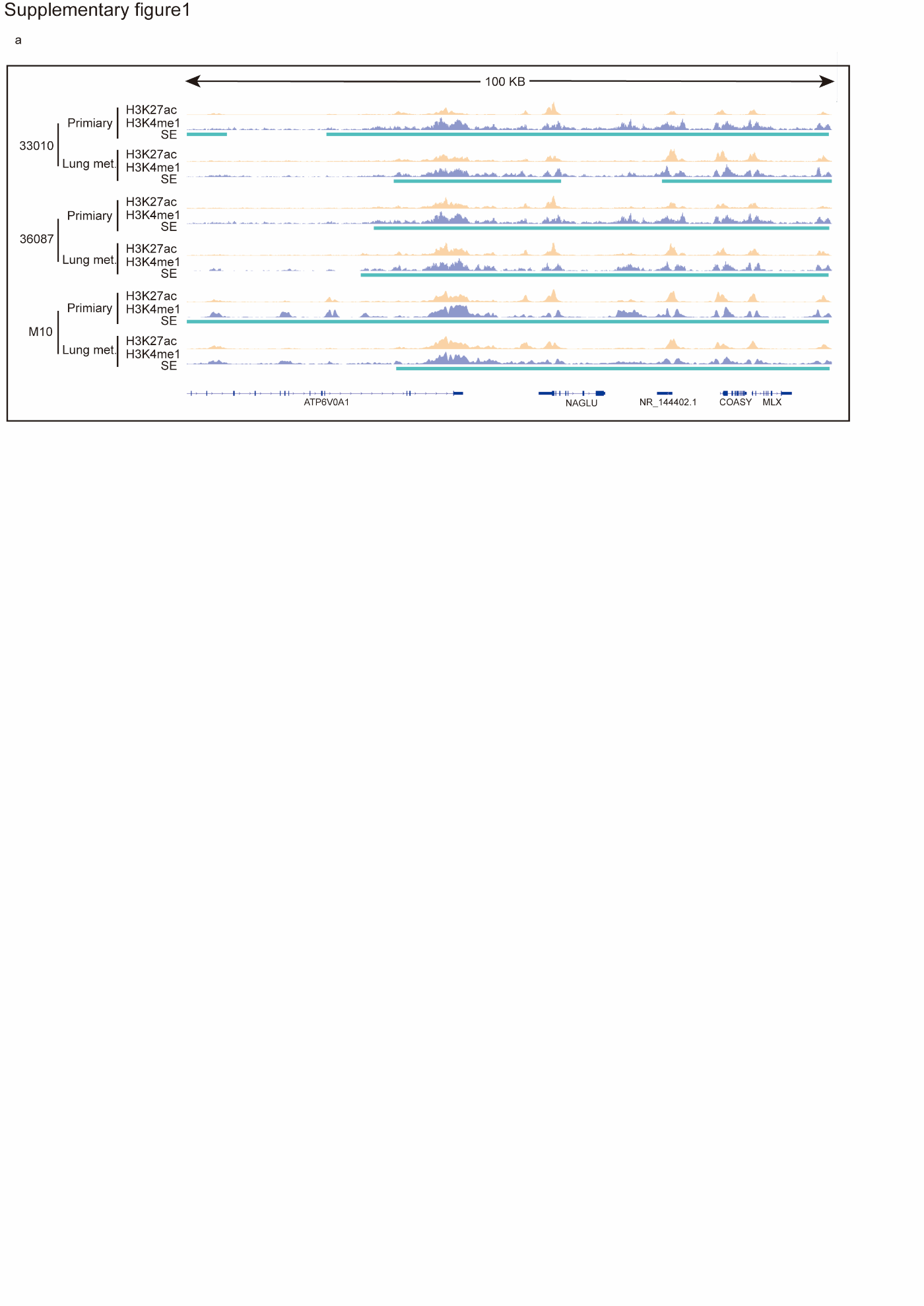
**

**Fig. S1** Super-enhancers (SEs) drive MLX expression in osteosarcoma. a) ChIP-seq binding profiles of H3K27ac and H3K4me1 in three pairs of clinical osteosarcoma samples (data from GSE74230) showing the SE regions associated with MLX gene locus. Primary, primary tumor; Lung met, lung metastasis. The blue boxes indicate the SE regions.

**
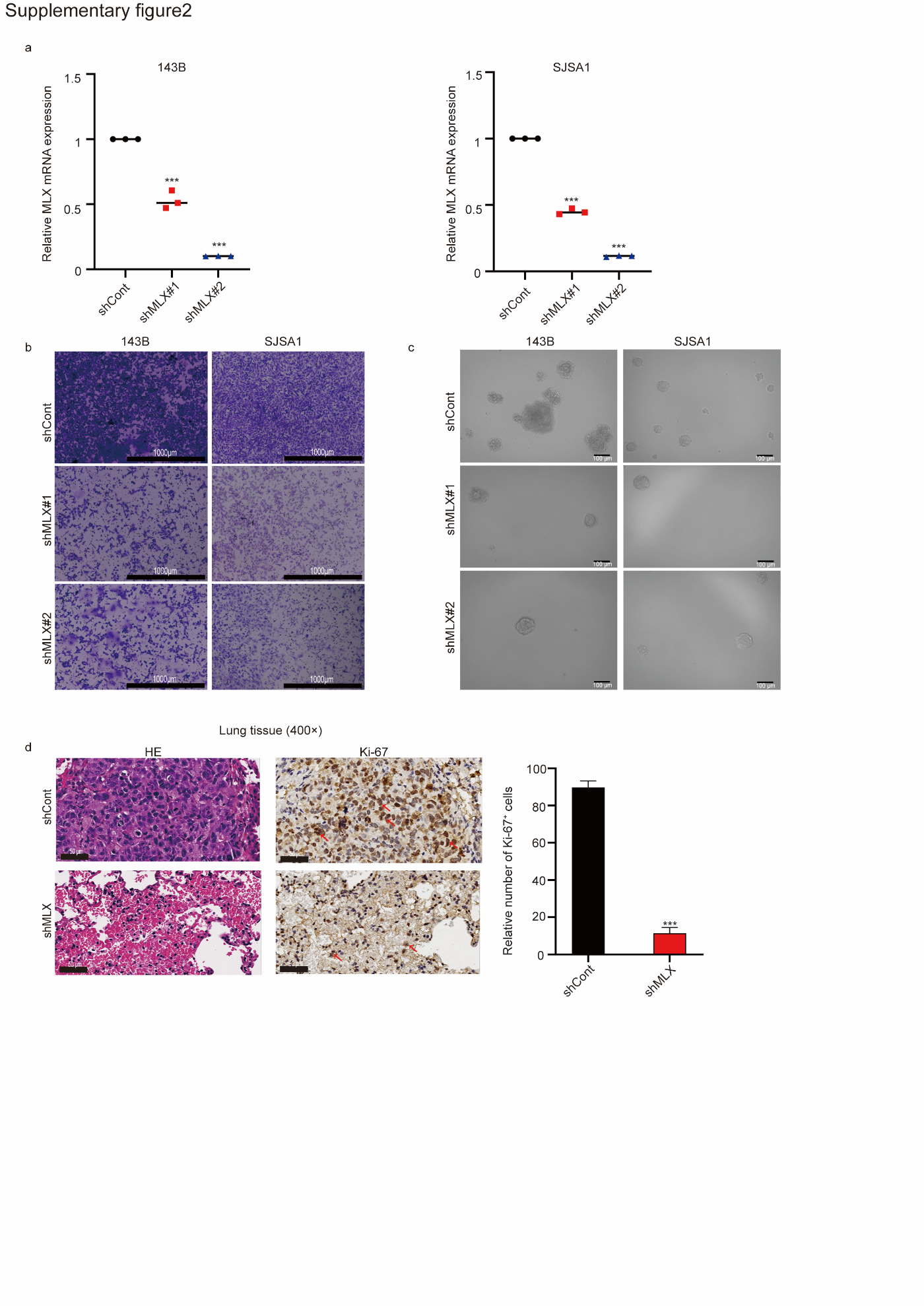
**

**Fig. S2** *MLX* knockdown inhibits invasion and sphere formation of osteosarcoma cells and reduces metastasis *in vivo*. a) RT-qPCR validation of *MLX* knockdown in 143B and SJSA1 cells. Data are represented as mean ± SD, n=3. Unpaired t-test was used. b) Representative images of the invasion assay in 143B and SJSA1 cells after *MLX* knockdown. Scale bar = 1000 μm. c) Representative images of the sphere formation assay in 143B and SJSA1 cells after *MLX* knockdown. Scale bar = 100 μm. d) Representative images of H&E and Ki-67 staining of lung tissues from 143B-shCont and 143B-sh*MLX* mice, and the ratios of Ki-67-positive cells in lung metastatic tissues. Scale bar = 50 μm. Data are represented as mean ± SD, n=3. Unpaired t-test was used. ***: P≤0.001, **: P≤0.01, *: P≤0.05, n.s.: not significant.


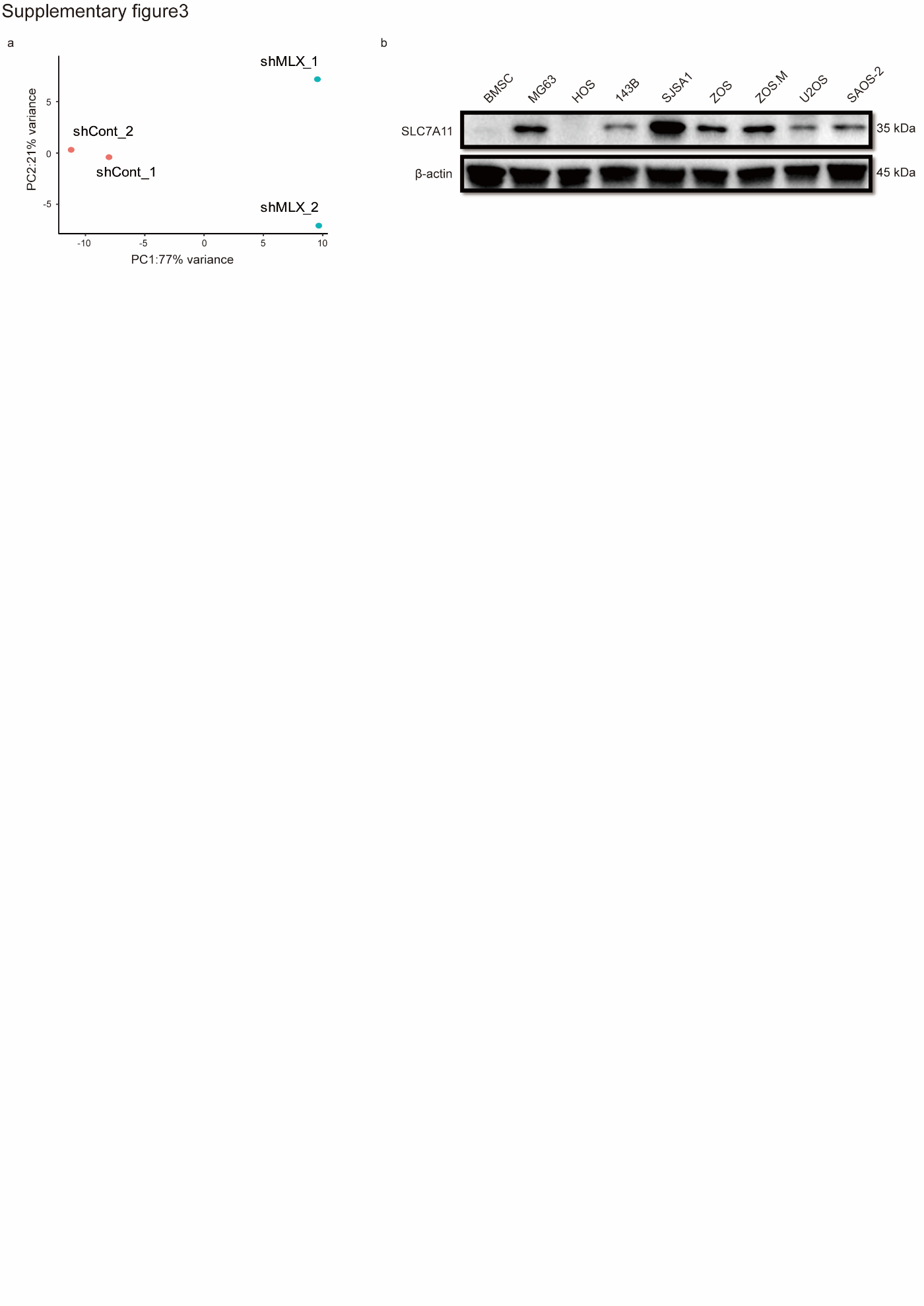


**Fig. S3** Silencing *MLX* caused significant gene expression changes. a) Principal component analysis of *MLX* knockdown cell transcriptome data shows clear separation between control and *MLX* knockdown cells, indicating significant changes in gene expression. b) Western blot analysis of SLC7A11 protein expression in cells (BMSC and osteosarcoma cells).


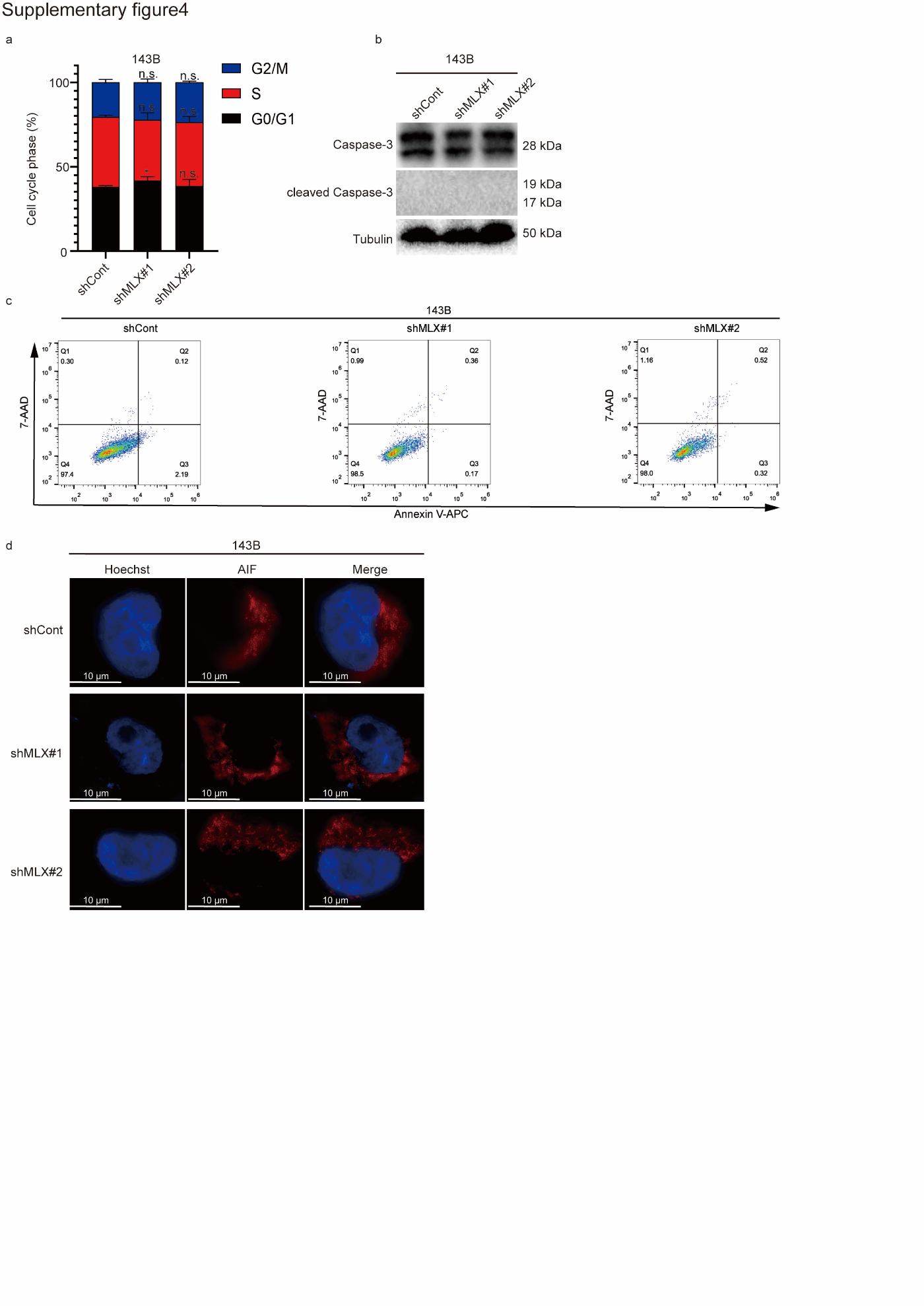


**Fig. S4** *MLX* knockdown induces cell death through ferroptosis. a) Cell cycle analysis of 143B after *MLX* knockdown. Data are represented as mean ± SD, n=3. Unpaired one-way ANOVA test followed by Dunnett’s test was used. b) The protein level of caspase-3 and cleaved caspase-3 in 143B upon *MLX* knockdown was detected by western blot (WB). c) Apoptosis profile of 143B after *MLX* knockdown. d) Immunofluorescence staining shows the localization of apoptosis-inducing factor (AIF) in 143B upon *MLX* knockdown (Scale bar = 10 μm). ***: P≤0.001, **: P≤0.01, *: P≤0.05, n.s.: not significant.


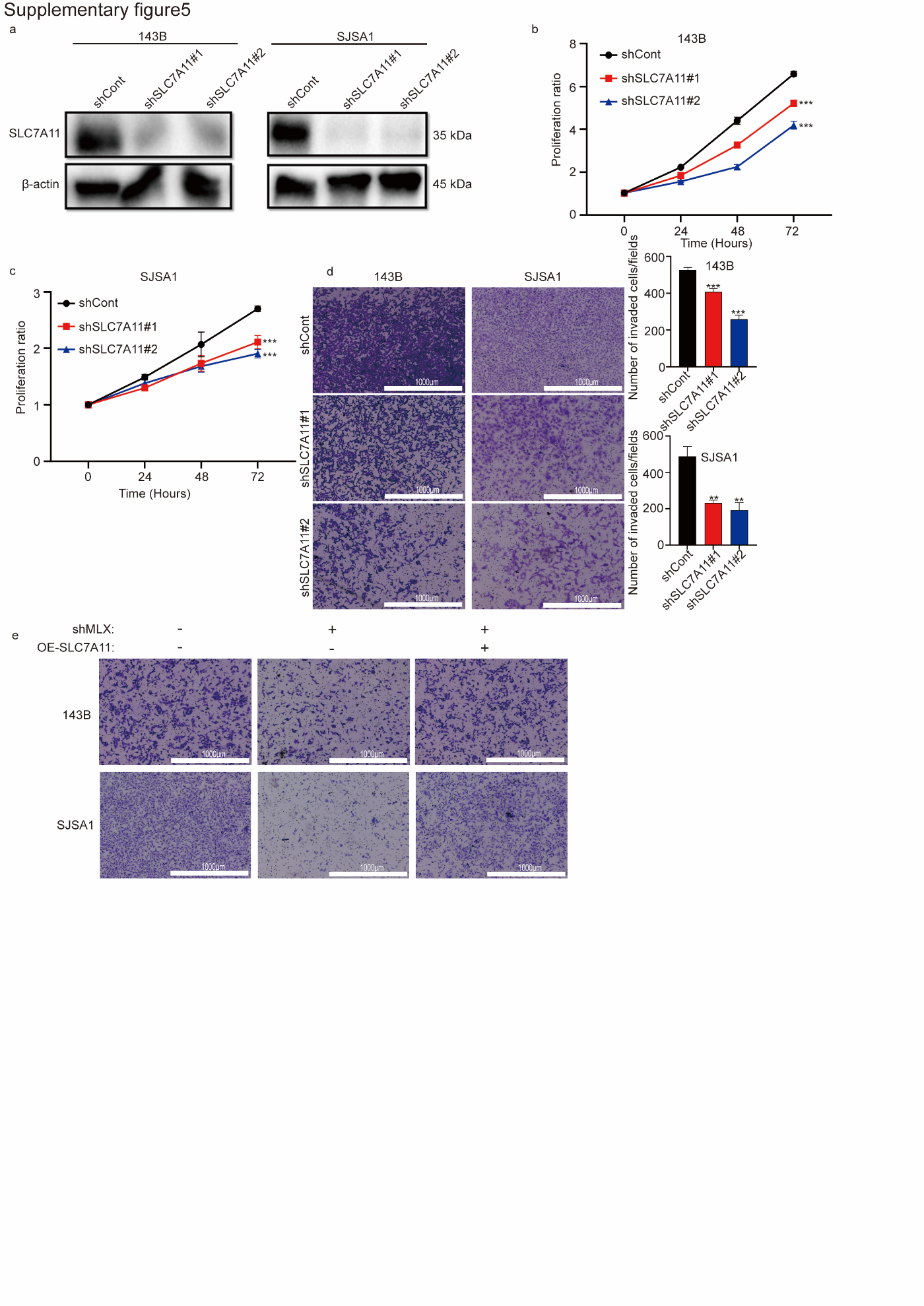


**Fig. S5** *SLC7A11* knockdown and overexpression affect the growth and invasion of osteosarcoma cells. a) *SLC7A11* knockdown in 143B and SJSA1 was confirmed by WB. b-c) Growth curves of 143B and SJSA1 cells stably transduced with scrambled control shRNA (shCont) or two SLC7A11 shRNAs (sh*SLC7A11*#1 and sh*SLC7A11*#2). Data are represented as mean ± SD, n=3. Unpaired one-way ANOVA test followed by Dunnett's test was used. d) Invasion assays were performed in sh*SLC7A11* versus shCont in 143B (left panel) and SJSA1 (right panel) cells. The number of migrated cells after 24 h was counted. Data are represented as mean ± SD, n=3. Unpaired t-test was used. (Scale bar = 1000 μm). e) Representative images of invasion assay in sh*MLX*-transfected 143B and SJSA1 cells overexpressing *SLC7A11* (Scale bar = 1000 μm). ***: P≤0.001, **: P≤0.01, *: P≤0.05, n.s.: not significant.


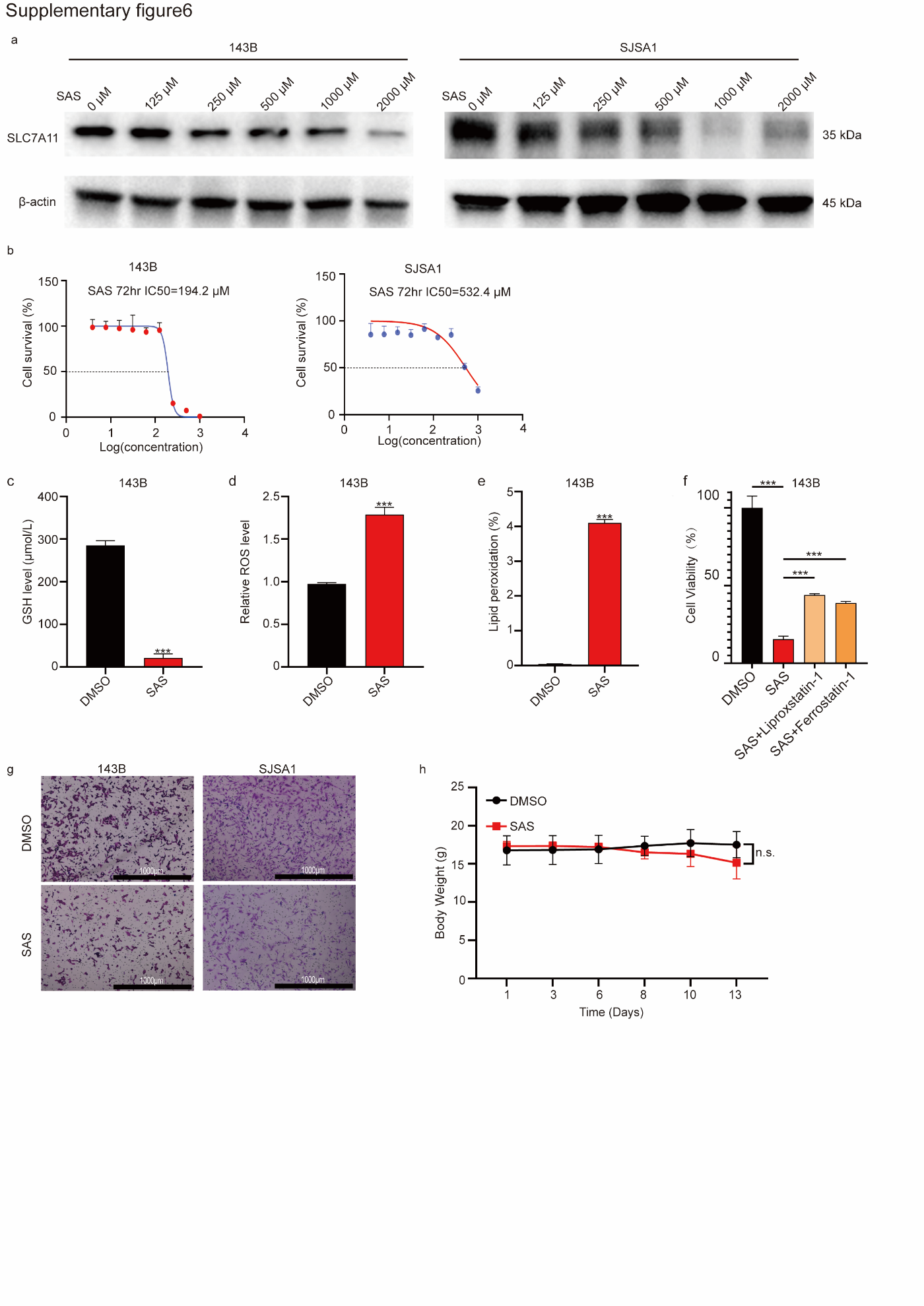


**Fig. S6** Sulfasalazine effectively suppresses the growth and metastasis of osteosarcoma in vitro and in PDXs. a) SLC7A11 protein expression in 143B and SJSA1 treated with sulfasalazine was detected by WB. b) 72-hour IC50 of sulfasalazine in 143B and SJSA1. c-e) Intracellular GSH (c), ROS (d), and lipid peroxidation levels (e) measured by flow cytometry in osteosarcoma cells treated with SAS. f) 24-hour cell viability of osteosarcoma cells treated with SAS and ferroptosis inhibitors (10 μM Liproxstatin-1 and 10 μM Ferrosatin-1). Data are represented as mean ± SD, n=3. Unpaired t-test was used. g) Representative images of invasion assay in 143B treated with DMSO and sulfasalazine (Scale bar = 1000 μm). h) The body weight curve of PDXs treated with DMSO or SAS. Data are represented as mean ± SD, n=5. Paired t-test was used. ***: P≤0.001, **: P≤0.01, *: P≤0.05, n.s.: not significant.

**Supplemental tables**

**Table S1** Sequences of shRNAs used in this study.

| Name | Direction | Sequence (5′-3′) |
| --- | --- | --- |
| shMLX#1 | Forward | CCGGGAGAGGCTATGATGACCTTCACTCGAGTGAAGGTCATCATAGCCTCTCTTTTTG |
|  | Reverse | AATTCAAAAAGAGAGGCTATGATGACCTTCACTCGAGTGAAGGTCATCATAGCCTCTC |
| shMLX#2 | Forward | CCGGGCAAGGATGTCACCGCCCTAACTCGAGTTAGGGCGGTGACATCCTTGCTTTTTG |
|  | Reverse | AATTCAAAAAGCAAGGATGTCACCGCCCTAACTCGAGTTAGGGCGGTGACATCCTTGC |
| shSLC7A11#1 | Forward | CCGGCCTGTCACTATTTGGAGCTTTCTCGAGAAAGCTCCAAATAGTGACAGGTTTTTG |
|  | Reverse | AATTCAAAAACCTGTCACTATTTGGAGCTTTCTCGAGAAAGCTCCAAATAGTGACAGG |
| shSLC7A11#2 | Forward | CCGGCCCTGGAGTTATGCAGCTAATCTCGAGATTAGCTGCATAACTCCAGGGTTTTTG |
|  | Reverse | AATTCAAAAACCCTGGAGTTATGCAGCTAATCTCGAGATTAGCTGCATAACTCCAGGG |
| shCont | Forward | CCGGCCTAAGGTTAAGTCGCCCTCGCTCGAGCGAGGGCGACTTAACCTTAGGTTTTTG |
|  | Reverse | AATTCAAAAACCTAAGGTTAAGTCGCCCTCGCTCGAGCGAGGGCGACTTAACCTTAGG |

**Table S2** Primers used in this study.

| Name | Direction | Sequence (5′-3′) |
| --- | --- | --- |
| MLX | Forward | ACAAGGAGTCCTACAAAGACCG |
|  | Reverse | CTTGCGTAACGTGGACACCT |
| GADPH | Forward | GCACCGTCAAGGCTGAGAAC |
|  | Reverse | ATGGTGGTGAAGACGCCAGT |
| SLC7A11 | Forward | TGACTGGAGTCCCTGCGTAT |
|  | Reverse | TGTTCTGGTTATTTTCTCCGACATT |
| ChIP-E1 | Forward | ATGGCTCACTGCAGCCTC |
|  | Reverse | CTGGTTATGATGGCAAATGCCTG |
| ChIP-E2 | Forward | AGACACTAAGGAGTGAGCGGTC |
|  | Reverse | AGGTAACTGCCCACACATATTTCAAG |
| ChIP-Negative control | Forward | TTAAATCTCTGGGAAGGTCTGTTCCG |
|  | Reverse | GCTTGTTGCTCAACTGACCTAAGC |
