# Supplementary material for: Super-enhancer-driven MLX mediates redox balance maintenance via SLC7A11 in osteosarcoma
Source: Cell Death Dis. 2023 Jul 17;14(7):439. doi: 10.1038/s41419-023-05966-y (PMC10352384; doi:10.1038/s41419-023-05966-y)

Uncropped images for Fig. 1D (MLX)

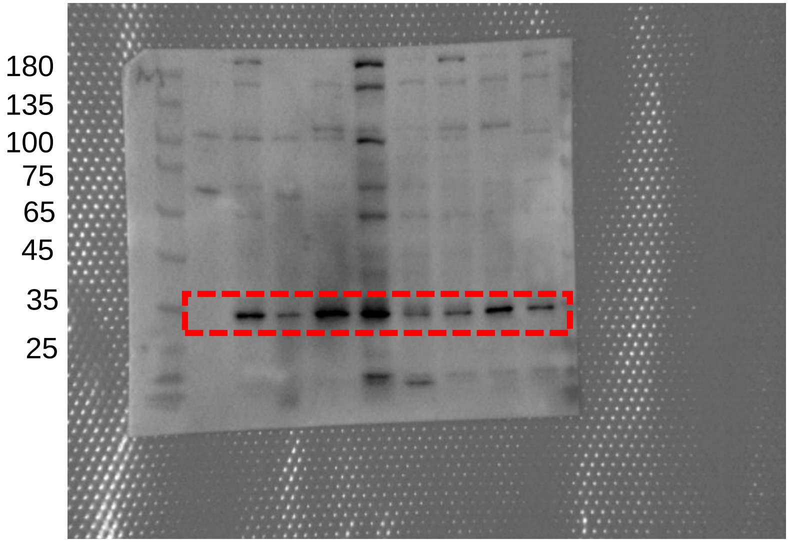

Uncropped images for Fig. 1D (β-actin)

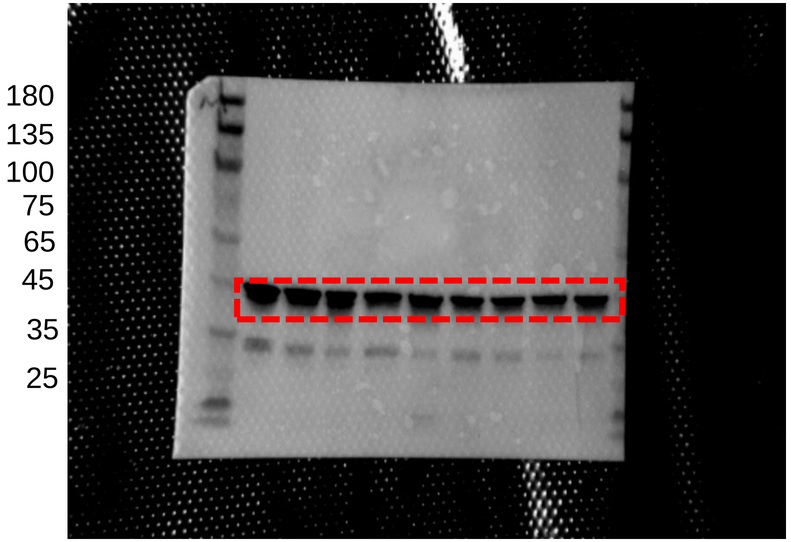

Uncropped images for Fig. 3D (SLC7A11 and Tubulin in 143B)

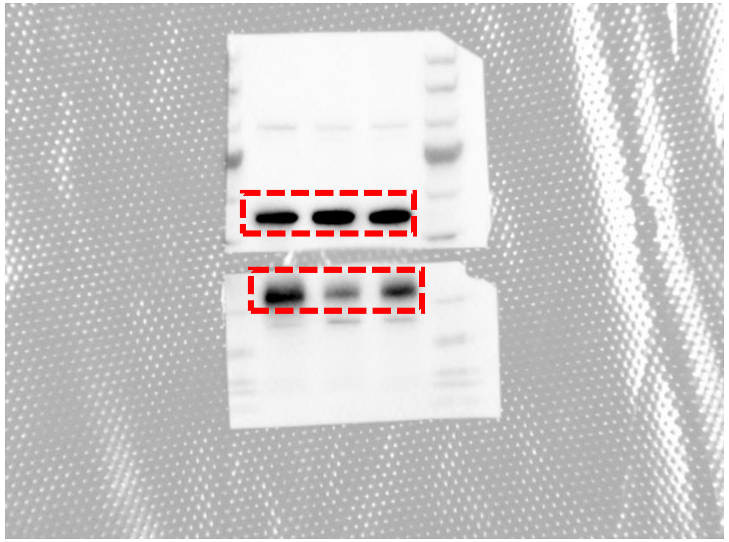

Uncropped images for Fig. 3D (SLC7A11 and Tubulin in SJSA1)

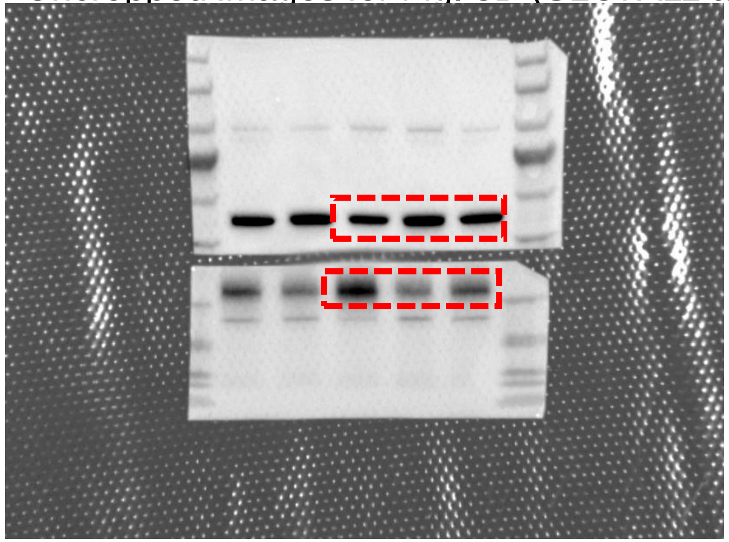

Uncropped images for Fig. S3b (SLC7A11)

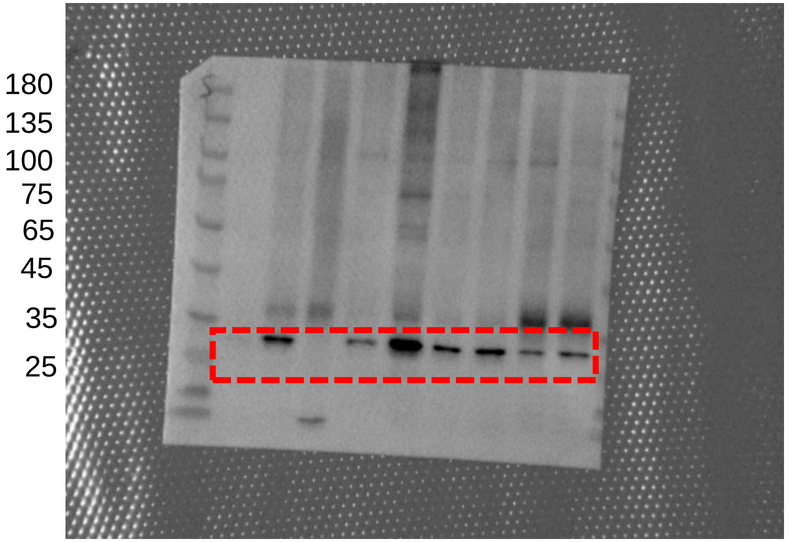

Uncropped images for Fig. S3b (β-actin)

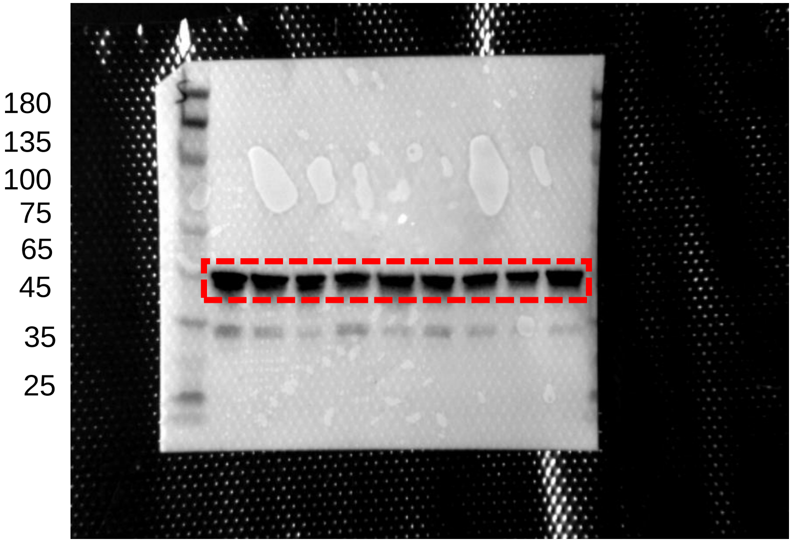

Uncropped images for Fig. 4E (MLX in 143B)

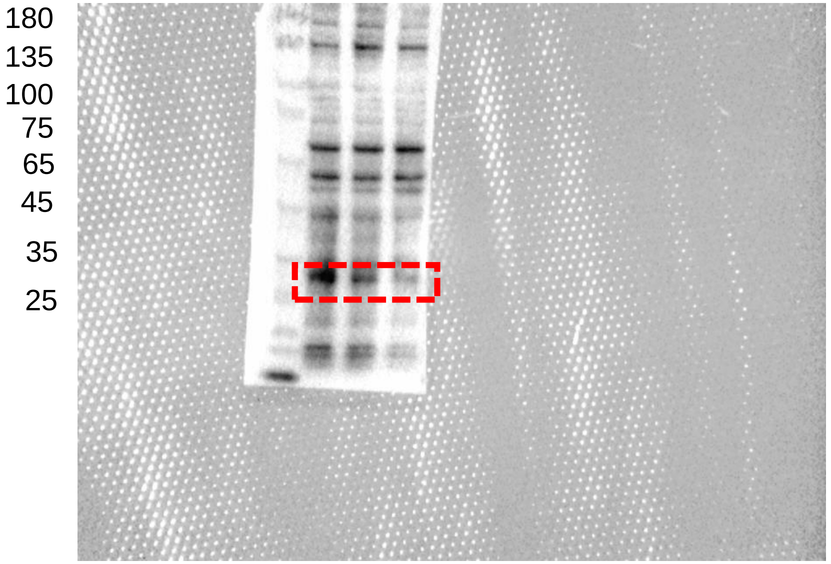

Uncropped images for Fig. 4E (4-HNE in 143B)

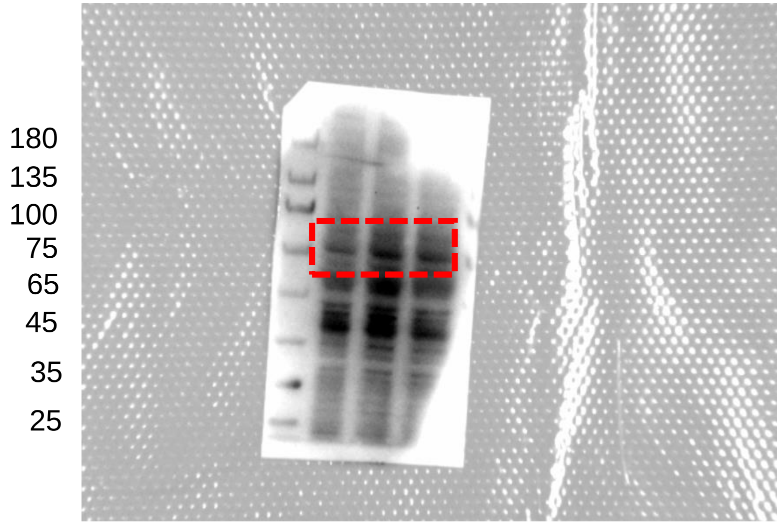

Uncropped images for Fig. 4E (β-actin in 143B)

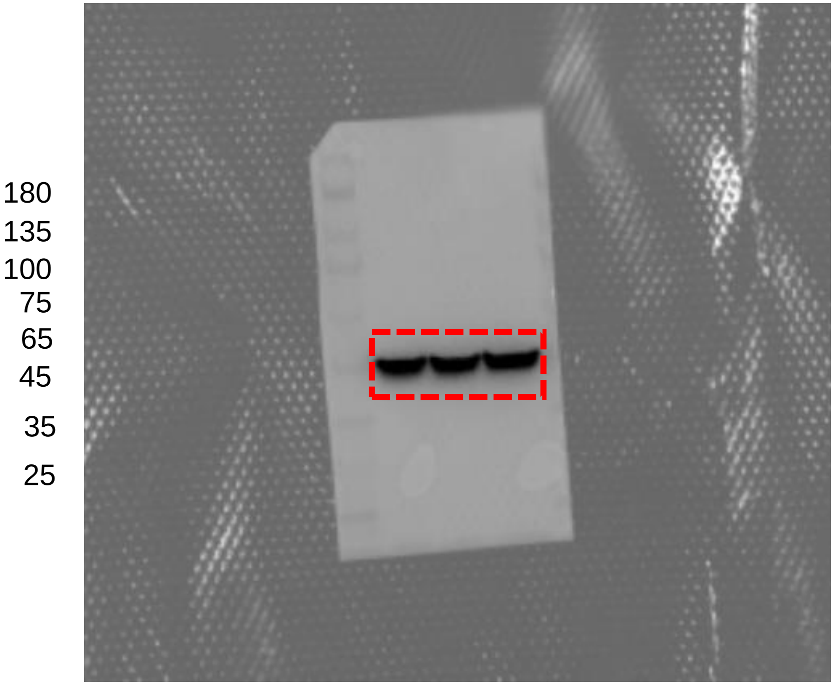

Uncropped images for Fig. 4F (CD71/ TRC、Transferrin 、SLC40A1/FPN1、 FTH1 and β-actin in 143B)

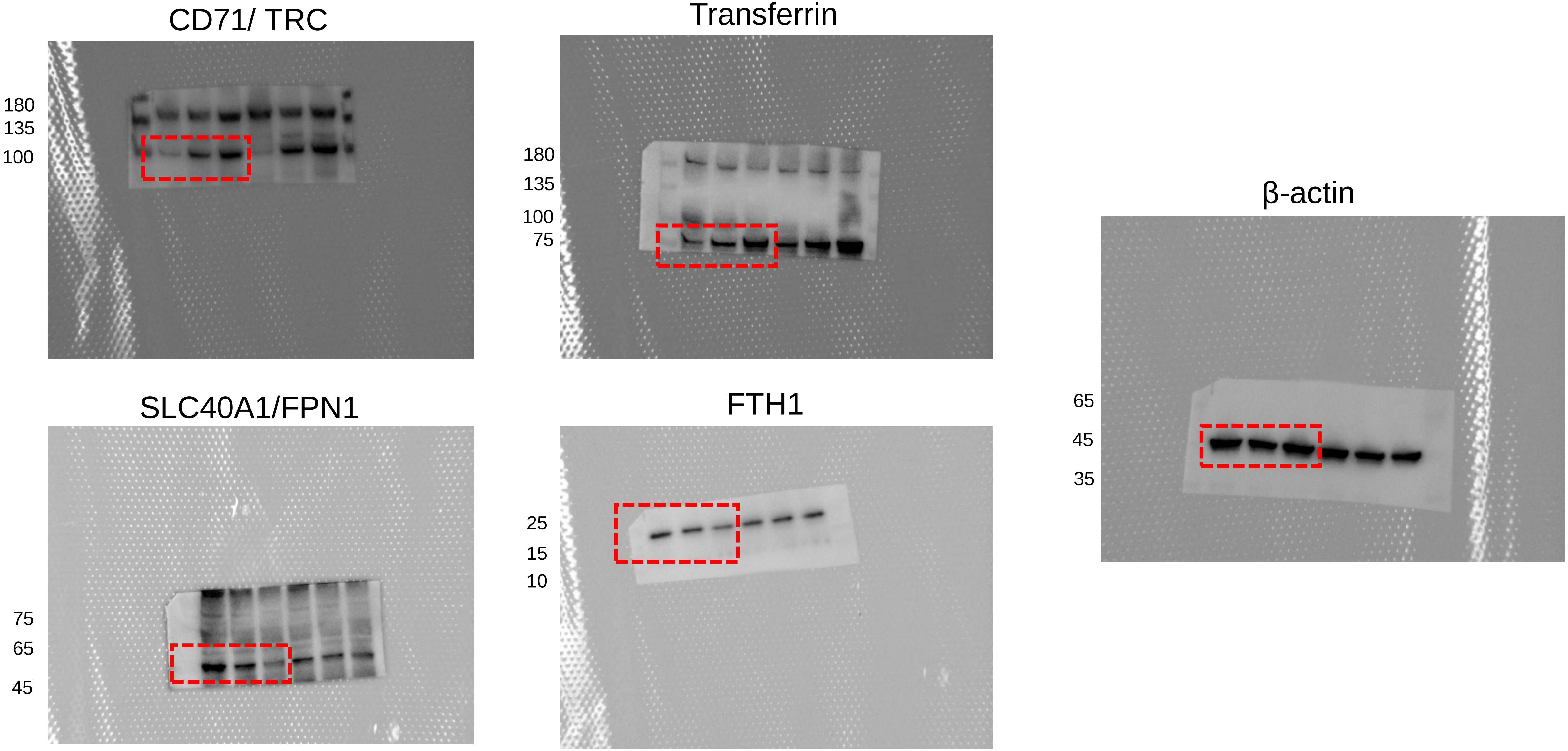

Uncropped images for Fig. S4b (Tubulin、Caspase-3 and cleaved caspase-3 in 143B)

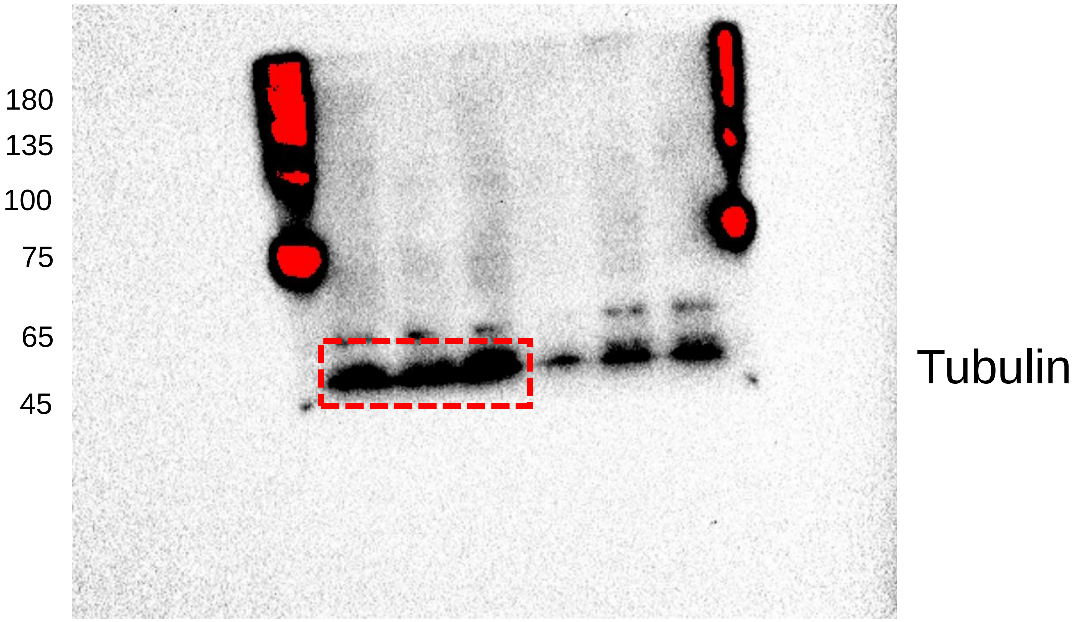

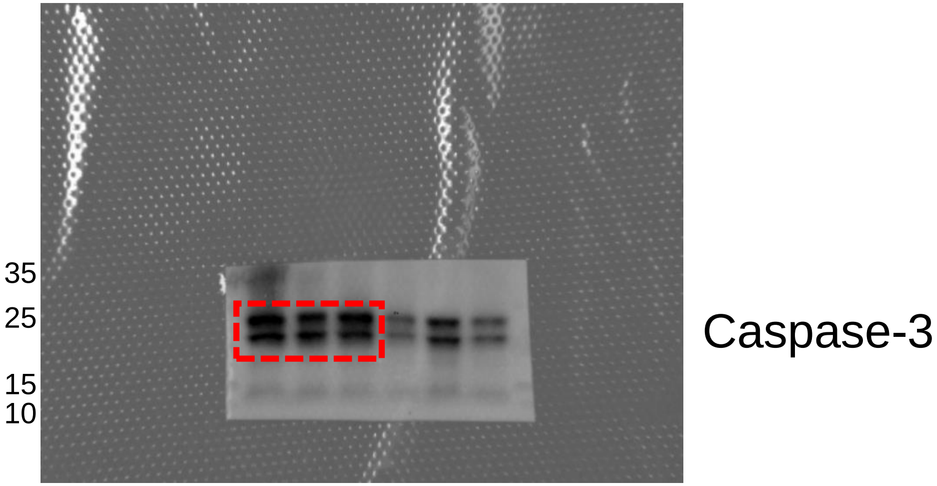

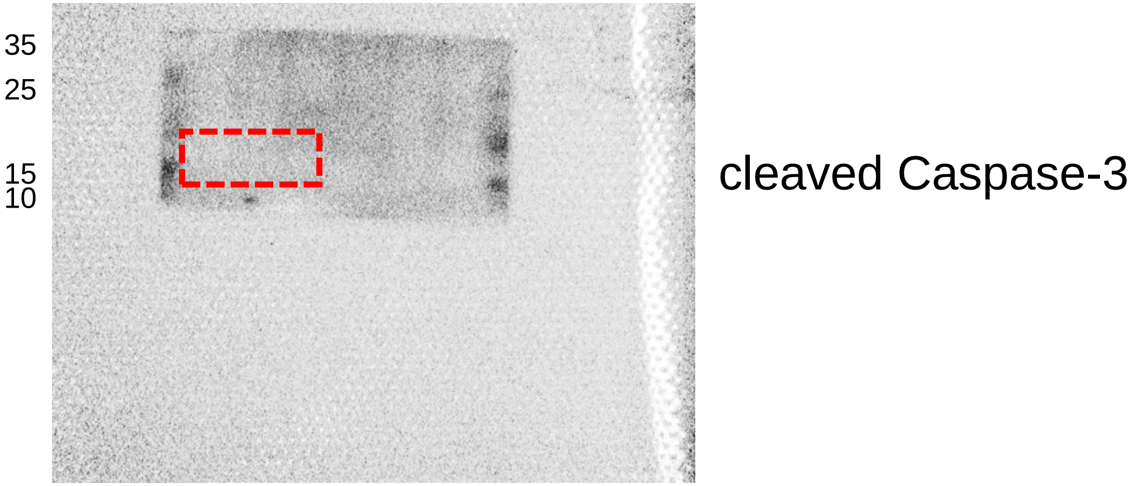

Uncropped images for Fig. 5A (SLC7A11 in 143B)

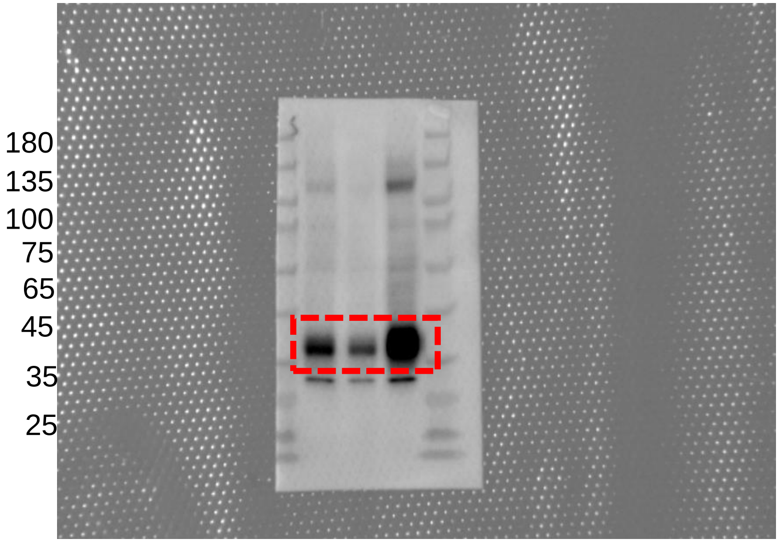

Uncropped images for Fig. 5A (MLX in 143B)

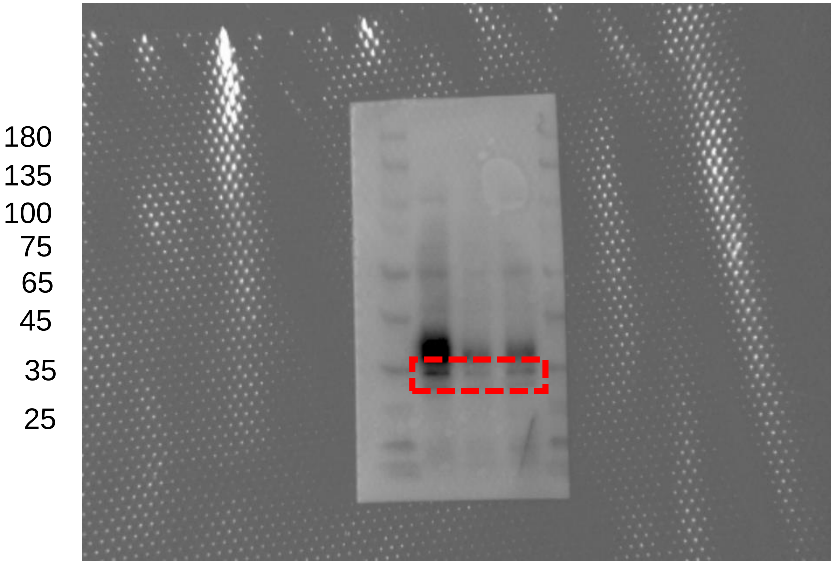

Uncropped images for Fig. 5A (β-actin in 143B)

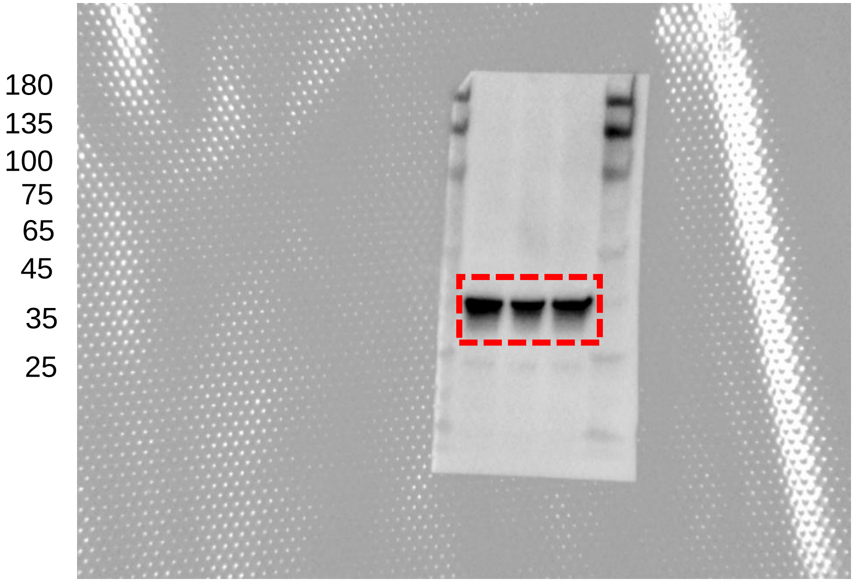

Uncropped images for Fig. 5A (SLC7A11 in SJSA1)

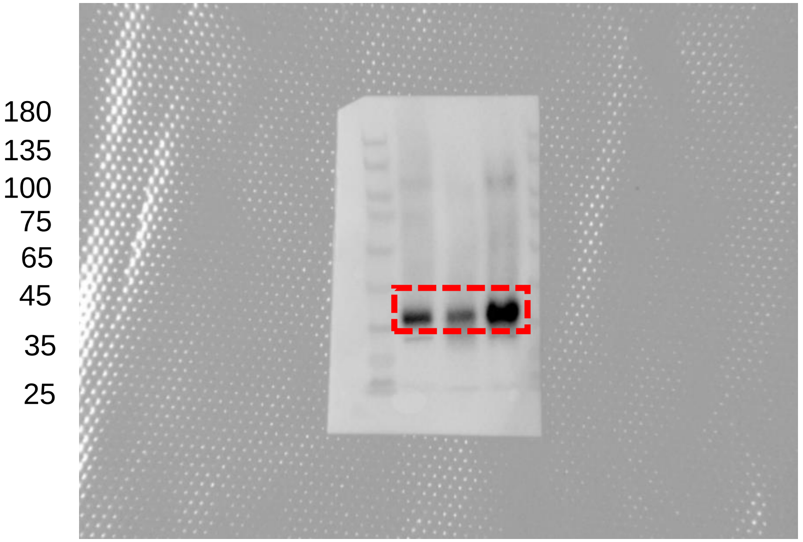

Uncropped images for Fig. 5A (MLX in SJSA1)

180
135
100
75
65
45
35
25

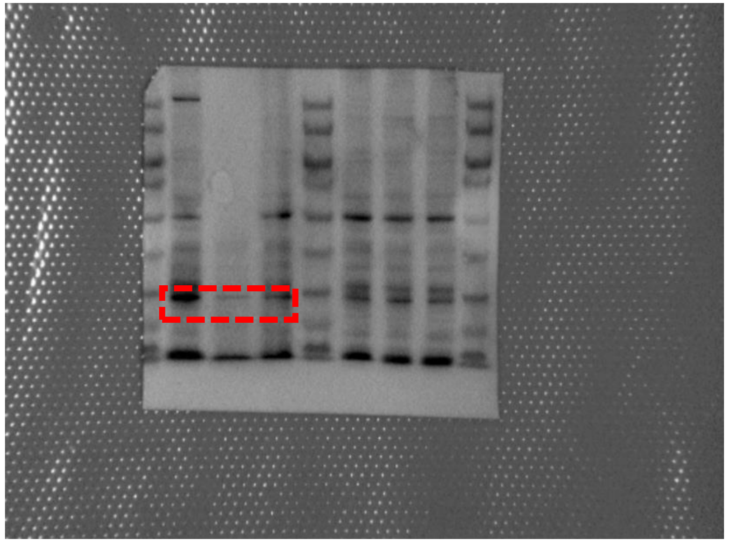

Uncropped images for Fig. 5A (β-actin in SJSA1)

180
135
100
75
65
45
35
25

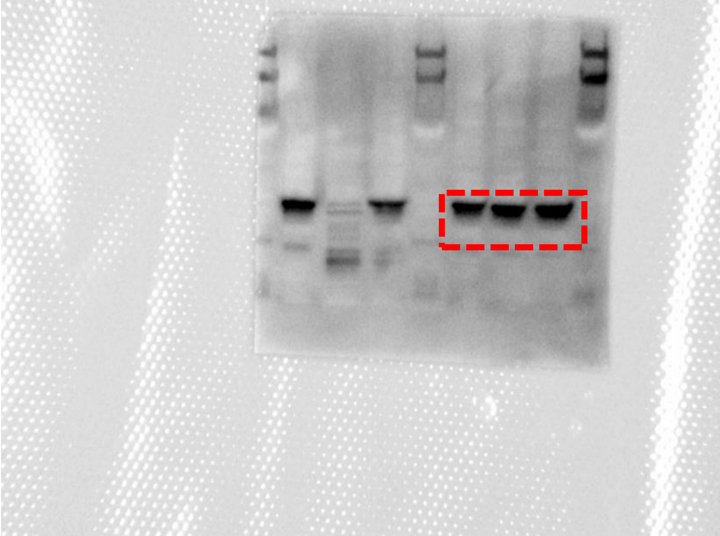

Uncropped images for Fig. S5a (SLC7A11 in 143B)

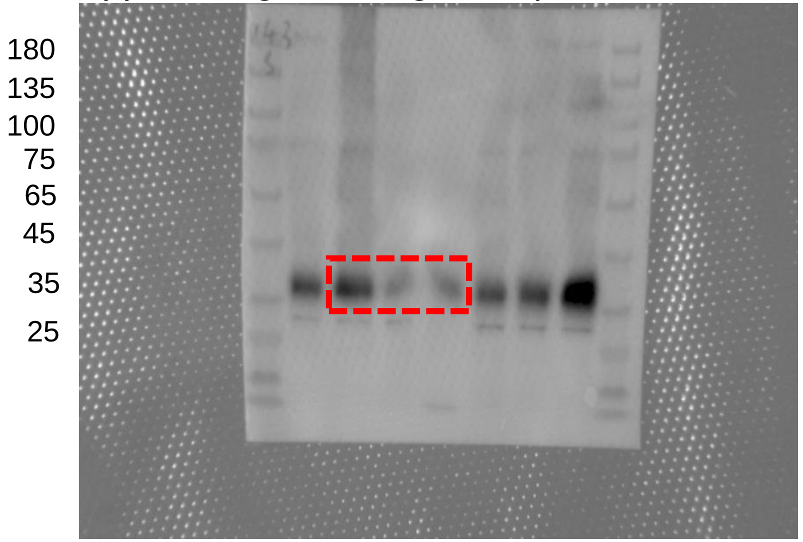

Uncropped images for Fig. S5a (SLC7A11 in SJSA1)

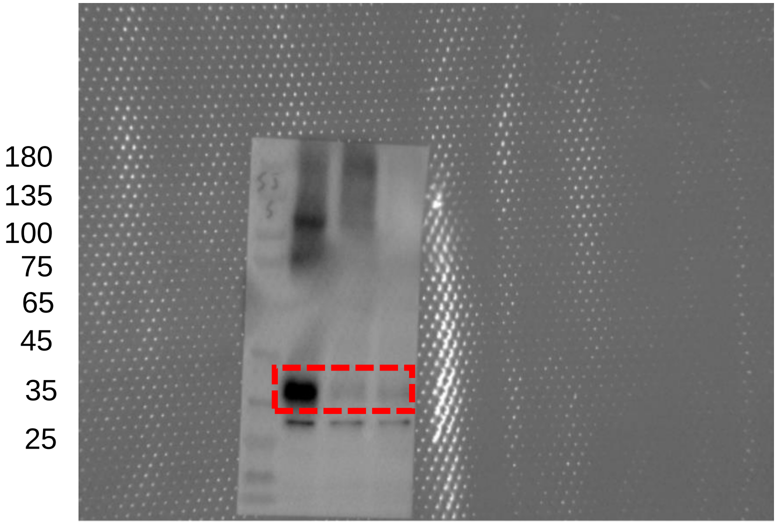

Uncropped images for Fig. S5a (β-actin in 143B)

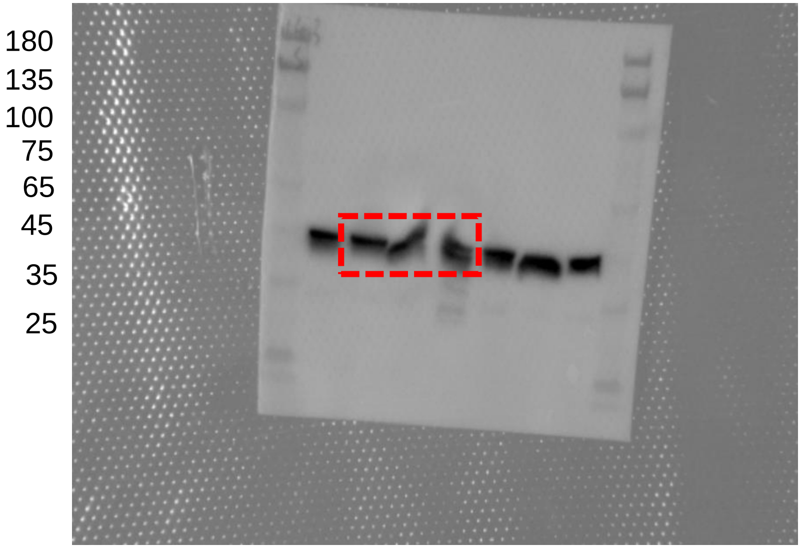

Uncropped images for Fig. S5a (β-actin in SJSA1)

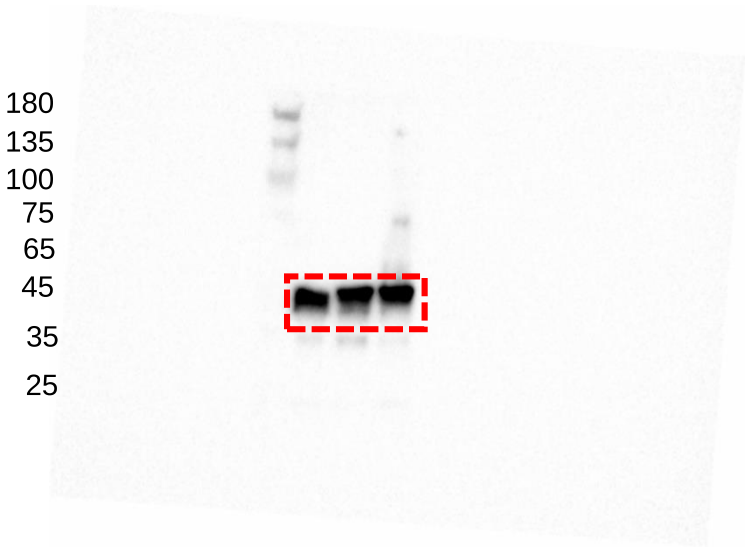

Supplement: Supplementary file 4 — Full and uncropped western blots [file 41419_2023_5966_MOESM4_ESM.pdf]
